# One Health at Risk: Plasmid-Mediated Spread of *mcr-1* Across Clinical, Agricultural, and Environmental Ecosystems

**DOI:** 10.3390/antibiotics14050506

**Published:** 2025-05-15

**Authors:** Abdelaziz Touati, Nasir Adam Ibrahim, Assia Mairi, Hassina Kirat, Nosiba S. Basher, Takfarinas Idres

**Affiliations:** 1 Laboratory of Microbial Ecology, Faculté Des Sciences de la Nature Et de la Vie (FSNV), University of Bejaia, Bejaia 06000, Algeria; abdelaziz.touati@univ-bejaia.dz (A.T.); assia.mairi@univ-bejaia.dz (A.M.); 2 Department of Biology, College of Science, Imam Mohammad Ibn Saud Islamic University (IMSIU), Riyadh 13318, Saudi Arabia; naabdalneim@imamu.edu.sa (N.A.I.); nsbasher@imamu.edu.sa (N.S.B.); 3 Laboratoire de Recherche des Interactions, Biodiversité, Ecosystèmes et Biotechnologie, Faculté des Sciences, Department of Natural and Life Sciences, University 20 August 1955—Skikda, Skikda 21000, Algeria; hasnakrt17@gmail.com; 4 Laboratory for Livestock Animal Production and Health Research, Rabie Bouchama National Veterinary School of Algiers, Issad ABBAS Street, BP 161 Oued Smar, Algiers 16059, Algeria

**Keywords:** plasmid-mediated resistance, *mcr-1* gene, colistin resistance, IncI2 plasmids, IncHI2 plasmids, IncX4 plasmids, one health

## Abstract

The global dissemination of plasmid-mediated *mcr* genes, which confer resistance to the last-resort antibiotic colistin, represents a critical public health challenge driven by the interplay of clinical, agricultural, and environmental factors. This review examines the genetic and ecological dynamics of *mcr*-bearing plasmids, focusing on their role in disseminating colistin resistance across diverse bacterial hosts and ecosystems. Key plasmid families demonstrate distinct evolutionary strategies, including IncI2, IncHI2, and IncX4. IncI2 plasmids favor stability in livestock and clinical settings. IncHI2 plasmids, on the other hand, leverage transposons to co-select for multidrug resistance, while IncX4 plasmids achieve global dissemination through streamlined, conjugation-efficient architectures. The pervasive spread of *mcr* genes is exacerbated by their integration into chromosomes via mobile genetic elements and co-selection with resistance to other antibiotic classes, amplifying multidrug-resistant phenotypes. Environmental reservoirs, food chains, and anthropogenic practices further facilitate cross-niche transmission, underscoring the interconnectedness of resistance under the One Health framework. Addressing this crisis requires coordinated strategies, including reducing colistin misuse in agriculture, enhancing surveillance of high-risk plasmid types, and fostering international collaboration to preserve antimicrobial efficacy and mitigate the threat of untreatable infections.

## 1. Introduction

Colistin, also known as polymyxin E, was first discovered in the 1940s and introduced for clinical use in the 1950s, but its early promise was curtailed by its significant nephrotoxicity and neurotoxicity [1]. With the emergence of multidrug-resistant (MDR) and extensively drug-resistant (XDR) Gram-negative bacteria, colistin has been globally re-introduced as a last-resort antibiotic to treat infections caused by pathogens such as *Pseudomonas aeruginosa*, *Acinetobacter baumannii*, and *Klebsiella pneumoniae* [2,3]. Its use has expanded in clinical and veterinary settings despite a narrow therapeutic index and the need for strict monitoring of plasma levels to avoid toxicity [4,5].

In agriculture, colistin has been extensively used in livestock farming, particularly for pigs and poultry, where it has been applied as both a prophylactic treatment and a growth promoter to prevent and control infections caused by enteric pathogens, such as *Escherichia coli* and *Salmonella* [6,7]. This widespread use in animal husbandry has contributed to the emergence and dissemination of colistin-resistant bacteria, as the routine application in food-producing animals creates an environment that fosters the selection and spread of resistance mechanisms [8]. These concerns have prompted regulatory efforts in various countries to restrict colistin usage in agriculture [5,9].

The reintroduction of colistin in clinical settings during the late 1990s and early 2000s was driven by the lack of effective alternatives to combat infections caused by carbapenem-resistant and other MDR Gram-negative bacteria. Despite its associated toxicities, colistin remains indispensable in intensive care units where life-threatening infections demand urgent intervention, often in combination with other antibiotics to mitigate adverse effects and enhance efficacy. This strategic reintroduction highlights colistin’s crucial role as a last-resort treatment when other therapeutic options have failed [10,11,12].

Colistin resistance arises through a combination of natural and acquired mechanisms. Intrinsic resistance is observed in certain Gram-negative species with inherent lipopolysaccharide (LPS) structure modifications, reducing colistin binding. More commonly, acquired resistance develops through chromosomal mutations in regulatory systems, such as *phoP*/*phoQ* and *pmrA*/*pmrB*, and the inactivation of genes like *mgrB*, resulting in LPS modifications (e.g., the addition of phosphoethanolamine or 4-amino-4-deoxy-L-arabinose) that decrease the antibiotic’s binding affinity. In addition, plasmid-mediated mechanisms—most notably the acquisition of *mcr* genes—facilitate the horizontal transfer of resistance determinants among bacterial populations, further complicating treatment strategies [1,13,14].

A pivotal moment in the history of colistin resistance was the first report of the *mcr-1* gene in 2015 in *E. coli* isolates from pigs in China [15]. This gene encodes a phosphoethanolamine transferase that modifies the lipid A portion of LPS, thereby reducing colistin binding and conferring resistance [16,17]. The discovery of *mcr-1* provided the first clear evidence of plasmid-mediated colistin resistance, alerting the scientific and medical communities to the potential for rapid global dissemination via horizontal gene transfer (HGT).

Following the identification of *mcr-1*, subsequent research has revealed a diverse array of *mcr* gene variants, ranging from *mcr-2* to *mcr-10*. Although these genes encode enzymes with a similar function—namely, the addition of phosphoethanolamine to lipid A—they differ in their nucleotide sequences, geographic distribution, and host range [18]. This genetic diversity underscores the adaptability of colistin resistance mechanisms and complicates efforts to monitor and control their spread across different bacterial species and regions [19,20].

The resistance conferred by *mcr* genes manifests as significantly elevated minimum inhibitory concentrations (MICs) for colistin [21]. By modifying the lipid A component of the bacterial outer membrane, the *mcr*-encoded enzymes reduce the net negative charge, thereby decreasing colistin’s binding affinity and subsequent bactericidal activity [16]. This plasmid-mediated resistance mechanism is particularly worrisome because it can be easily transferred between different bacterial species, often co-existing with other resistance determinants and leading to MDR phenotypes [22,23].

Colistin-resistant bacteria harboring *mcr* genes have been isolated from a broad spectrum of sources, including clinical specimens (such as bloodstream and urinary tract infections), agricultural products (including meat and dairy), and various environmental samples (e.g., wastewater and soil). The widespread detection of *mcr*-positive isolates in both human and animal sectors and environmental reservoirs illustrates colistin resistance’s extensive distribution and interconnectivity, further complicating efforts to control its spread [24,25,26].

The rapid global emergence of colistin resistance, driven by its overuse in clinical and agricultural settings and the spread of the plasmid-mediated *mcr* gene, poses a critical One Health threat, intertwining human, animal, and environmental health. Colistin’s eroding efficacy—a last-resort therapy for MDR and XDR Gram-negative infections—risks untreatable human infections, escalating mortality, and increased healthcare burdens [27]. Simultaneously, its non-therapeutic use in livestock, particularly in regions with weak oversight, selects for resistant bacteria in animals, which spread via food chains, occupational exposure, or environmental contamination [24,28]. Resistant pathogens and *mcr* genes continue to infiltrate ecosystems through agricultural runoff, wastewater, and improper disposal of antibiotics, becoming embedded in soil and water systems. These environmental reservoirs enable the persistent circulation of resistance genes and horizontal transfer, creating feedback loops that jeopardize wildlife and potentially recontaminate agricultural or human populations [26]. Addressing this crisis requires unified One Health strategies, including enforcing antibiotic stewardship in human and veterinary medicine, banning agricultural misuse, enhancing surveillance across all sectors, and adopting sustainable practices such as advanced wastewater treatment. Without such integrated interventions, the decline of colistin’s utility will accelerate the onset of a post-antibiotic era, underscoring the urgency of global, cross-sectoral collaboration [29,30].

The global spread of plasmid-borne colistin resistance, primarily mediated by *mcr* genes, poses a critical challenge to public health. This article synthesizes recent findings on genetic architectures, resistance profiles, and transmission dynamics of plasmids associated with *mcr* genes.

## 2. Global Dissemination of the *mcr* Gene

The global dissemination of the *mcr* gene, which confers resistance to the last-resort antibiotic colistin, underscores the interconnectedness of human, animal, and environmental health, as emphasized by the One Health framework. The compiled data from Table S1 (Supplementary Table S1) reveal its pervasive presence across diverse reservoirs, bacterial species, and geographic regions, highlighting a critical public health challenge.

In human clinical settings, *mcr* prevalence varies significantly, with notable rates in Argentina (4.2% in *E. coli* isolates) [31], Pakistan (66% in *K. pneumoniae* strains) [32], and China (2.8% in *E. coli* strains) [33]. Lower rates, such as 0.6% in Nepal [34] and 0% in Switzerland [35] suggest regional disparities in the emergence of resistance or surveillance sensitivity. Fecal carriage studies further demonstrate transmission risks, with high rates observed in Bolivia (38.3% among rural children) [36] and China (35.8% among healthy children) [37], in contrast to negligible detection in Dutch institutional residents [38].

Animal reservoirs serve as critical *mcr* reservoirs. Livestock in China exhibits an alarmingly high prevalence, with rates of 91% in food animals [39] and 98% in Portuguese pigs [40]. Wild and migratory species, including vampire bats in Peru (33%) [41], Père David’s deer in China (69.1%) [42], and Barbary macaques (1.2%) in Algeria [43], illustrate the gene’s spillover into wildlife. Poultry systems globally show variability, from 57.9% in Brazilian broilers [44] to 6.8% in Lebanese farms [45], reflecting differences in antimicrobial use or biosecurity.

The food chain is a key transmission route. Retail meats in Egypt (19%) [46], Japan (21%) [47], and the Netherlands (24.8%) [48] frequently harbor *mcr*-positive strains. Contaminated vegetables, although less common, have been sporadically detected, with rates as low as 0.42% in Chilean produce [49] and 0.5% in Algerian leafy greens [50]. Seafood in South Africa (90%) [51] and aquatic products in China (47.1% in crocodile cecum samples) [52] further highlight the risks associated with aquaculture.

Environmental compartments act as reservoirs and dissemination pathways. Wastewater in Germany (9.6%) [53] and China’s Haihe River (100%) [54] demonstrate widespread aquatic contamination. Agricultural and urban sewage systems, such as those in Spain (30 *mcr-1* strains), facilitate the persistence and spread of genes. Notably, *mcr* remains undetected in U.S. livestock and environmental sources [55], suggesting regional success in containment or gaps in surveillance.

Geographically, *mcr* spans six continents, with dense reporting in Asia and Europe. China’s multifaceted prevalence—from clinical (7.5% in hospitalized patients) [56] to environmental (100% in river samples) [54]—reflects its role as a hotspot. Conversely, regions such as the USA and Switzerland report minimal or no detection, suggesting variable selection pressures or ineffective stewardship [35,55].

## 3. Plasmids Harboring the *mcr* Gene

The emergence of plasmid-mediated colistin resistance, conferred by the *mcr* gene family, represents a critical threat to global public health. Among the diverse plasmid incompatibility (Inc) groups implicated in *mcr* dissemination, IncHI2, IncI2, and IncX4 stand out as dominant vectors, facilitating the spread of resistance across bacterial species, ecological niches, and geographical boundaries (Supplementary Table S2). The dominance of IncHI2, IncI2, and IncX4 plasmids is amplified by their ability to transcend species barriers. For instance, *mcr-1* on IncHI2 has been identified in both *E. coli* (human clinical isolates) and *Salmonella* (foodborne outbreaks), while IncX4 plasmids bridge human, animal, and environmental reservoirs (e.g., detected in pediatric patients, retail meat, and river water). Hybrid plasmids (e.g., IncHI2/IncN) further illustrate the plasticity of these vectors, though their prevalence remains secondary to the three major groups (Table 1).

### 3.1. Structural and Functional Overview of IncI2 Plasmids

IncI2 plasmids are a significant class of mobile genetic elements recognized for their ability to harbor the *mcr-1* gene, a crucial determinant of colistin resistance. These plasmids are predominantly found in *E. coli* and *Salmonella enterica* strains, with notable isolation from clinical and environmental sources, including human and animal clinical samples and wastewater and food environments [67,68]. The size of IncI2 plasmids typically ranges from 58 to 68 kb, making them relatively small compared to other plasmids [69,70]. These plasmids exhibit a GC content of between 42% and 45%, consistent with other Inc plasmid types, contributing to their stable replication and transmission [70,71].

The self-transmissibility of IncI2 plasmids is a defining characteristic, as they can transfer the *mcr-1* gene to other bacteria via conjugation, contributing significantly to the horizontal spread of colistin resistance [72,73]. This is particularly concerning in clinical infections, where *E. coli* strains carrying IncI2 plasmids are often associated with MDR phenotypes. The conjugative nature of these plasmids, coupled with their presence in various bacterial hosts, suggests a broad host range, which enhances their potential for interbacterial transfer and propagation of resistance [74,75]. In clinical settings, IncI2 plasmids have been identified in *E. coli* strains responsible for urinary tract infections, bloodstream infections, and other hospital-acquired infections [76,77]. The incidence of IncI2 plasmids in human clinical isolates highlights their potential role in spreading colistin resistance in healthcare-associated infections [78,79]. A study in China highlighted the presence of IncI2 plasmids in clinical *E. coli* strains isolated from urinary tract infections and biliary tract infections, which carry not only the *mcr-1* gene but also additional resistance genes such as *bla*_CTX-M_, contributing to extended-spectrum beta-lactamase (ESBL) resistance [80,81].

Moreover, IncI2 plasmids are not restricted to clinical environments but are widespread in animal populations, particularly poultry and swine [82,83]. In poultry farms, the IncI2 plasmids carry *mcr-1*. In some instances, they harbor additional resistance genes, including tetracycline resistance genes (*tet*(A)), and resistance to sulfonamides and aminoglycosides, resulting in strains with MDR profiles [84,85]. These findings underscore the significant role of animal reservoirs in transmitting colistin resistance to humans, particularly in regions with intensive agricultural practices and widespread antibiotic use [74,86].

Environmental sources, such as wastewater treatment plants, also serve as hotspots for disseminating IncI2 plasmids carrying the *mcr-1* gene. Plasmids have been identified in *E. coli* and *K. pneumoniae* strains isolated from wastewater [73,87]. These environmental isolates serve as critical vectors for colistin resistance in aquatic and soil ecosystems, thereby contributing to the global dissemination of the *mcr-1* [88].

Interestingly, IncI2 plasmids exhibit genetic diversity, with some displaying high sequence similarity to previously characterized IncI2 plasmids, such as pHNSHP45, which is considered a reference plasmid for *mcr-1*-bearing IncI2 plasmids [69,89]. Despite their genetic diversity, many IncI2 plasmids exhibit conserved backbone elements, including replication genes (*repA*), conjugation genes (*tra*, *pil*), and plasmid stability genes (*parA*), which enable their stable inheritance and efficient horizontal transfer [87,90].

Another notable feature of these plasmids is the persistence of the *mcr-1* gene, as many IncI2 plasmids are capable of stable maintenance even without selective pressure. For example, in studies of *E. coli* strains carrying IncI2 plasmids, plasmid stability remained high after multiple passages, suggesting that these plasmids are well-adapted for long-term persistence in bacterial populations [59,78]. This stability is crucial for the long-term dissemination of colistin resistance in clinical and environmental contexts, even without frequent antibiotic use.

The ability of IncI2 plasmids to harbor a broad array of resistance genes contributes to their role in MDR. IncI2 plasmids carrying the *mcr-1* gene are frequently found in conjunction with other antibiotic resistance genes, including those conferring resistance to beta-lactams (e.g., *bla*_TEM_, *bla*_CTX-M_), quinolones (e.g., *qnrS*), aminoglycosides, and tetracyclines [80,81,90,91]. This combination of colistin resistance and resistance to other antibiotics complicates treatment strategies. It is of significant concern in clinical settings, where infections caused by MDR pathogens are increasingly challenging to manage [76,92].

### 3.2. Structural and Functional Overview of IncHI2 Plasmids

IncHI2 plasmids are among the most prominent mobile genetic elements associated with disseminating *mcr* genes [93,94]. These plasmids are typically large, ranging from approximately 60 kb to over 310 kb, though most fall within the 200–280 range [80,95]. Even larger IncHI2 plasmids have been reported, such as the 298.6 kb pSal008 found in ready-to-eat pork [96] and a 310.1 kb plasmid in *Salmonella* Typhimurium from pediatric fecal samples [97]. The GC content of these plasmids typically averages 46–47%, as confirmed in several isolates [96,98].

A defining feature of IncHI2 plasmids is their conjugative nature, facilitated by a comprehensive suite of transfer-associated genes, including *tra*, *oriT*, and *pil* [90,99,100]. These genes support the efficient HGT of resistance determinants, including *mcr*, across diverse bacterial hosts. Conjugation experiments have demonstrated the successful transfer of IncHI2 plasmids to *E. coli*, *K. pneumoniae*, and *Salmonella* recipients, with conjugation frequencies ranging from 10^−6^ to 10^−4^, depending on the genetic context and environmental conditions [46,85,101].

These plasmids encode multiple functional modules critical for replication (e.g., *repA*), stability (e.g., *parA*), and maintenance, ensuring their persistence within host cells [90,100]. Multireplicon structures such as IncHI2/IncN, IncHI2A/IncHI2, and IncHI2/IncQ are common, enhancing compatibility with various host strains and plasmid systems [58,75,102].

Geographically, IncHI2 plasmids are widely distributed across Asia, Europe, Africa, and Latin America, appearing in isolates from clinical samples, animal production systems, wastewater, and food products [22,93,99,103]. They are often found in high-risk sequence types, such as ST34 in *Salmonella* Typhimurium, which is frequently associated with MDR profiles in clinical and foodborne isolates [85,104,105].

An additional trait of IncHI2 plasmids is their tendency to carry integrons, operons (e.g., tellurium resistance operon *terABCDE*), and transposons, which further contribute to their genetic plasticity [100]. Their ability to fuse with other replicon types (e.g., IncN, IncQ) to form hybrid plasmids with expanded resistance profiles has been documented [102,106,107], complicating containment and surveillance efforts.

The large size, broad host range, conjugative potential, and multi-replicon structure of IncHI2 plasmids make them highly efficient vectors for disseminating *mcr* genes and other resistance determinants. Their prevalence in clinical and agricultural environments highlights their pivotal role in the global spread of multidrug resistance [86,108,109].

### 3.3. Structural and Functional Overview of IncX4 Plasmids

IncX4 plasmids represent one of the most widespread and evolutionarily successful vehicles for disseminating the mobile colistin resistance gene *mcr-1* and, in fewer cases, *mcr-2*. These plasmids have been reported across multiple continents from various sources, including humans, animals, food, and the environment, underscoring their critical role in the One Health dissemination of colistin resistance [64,110,111,112].

A defining characteristic of IncX4 plasmids is their small and conserved size, typically ranging from ~29 to 60 kb, with most *mcr*-carrying IncX4 plasmids clustering between 32 and 34 kb [95,111,113,114]. The GC content of IncX4 plasmids typically falls within a narrow range (reported between 41.9–44.4%), and their coding capacity includes about 40–44 predicted ORFs, consistent with their compact and streamlined architecture [71,78,85,115]. Their genetic organization is highly conserved across bacterial species and geographical settings, with comparative genomics revealing greater than 99% sequence identity among IncX4 plasmids isolated in Brazil, China, Poland, and elsewhere [75,91,112].

A central feature of IncX4 plasmids is their high transferability. They are conjugative plasmids and often encode a Type IV secretion system (T4SS), essential for horizontal transfer across bacterial species [116]. Experimental conjugation assays have confirmed the successful transfer of IncX4 plasmids carrying *mcr-1* from *E. coli*, *K. pneumoniae*, and *P. aeruginosa* to laboratory recipient strains [61,95]. The plasmid from *P. aeruginosa* was confirmed to belong to the P-31 MOB subgroup, and its conjugative ability was retained even in clinical isolates co-harboring *bla*_NDM-1_ [110].

Another notable trait is the structural simplicity and high backbone stability of IncX4 plasmids. Unlike IncHI2 or IncI2 plasmids, which frequently contain insertion sequences (e.g., IS*Apl1*) and transposons, IncX4 plasmids often lack such mobile elements, resulting in fewer structural rearrangements and more stable integration of the *mcr-1* gene [25,117,118]. This absence of mobile elements may reduce the likelihood of transposition but enhances the persistence of the resistance gene in the plasmid backbone, even without selection pressure [46,59,119]. Indeed, a study from the Czech Republic found that *E. coli* isolates exhibited inactivation of *mcr-1* due to IS2 insertion, which was reversible under colistin exposure, demonstrating that IncX4 backbones are less prone to disruption unless under intense selective pressure [120].

Although early studies reported IncX4 plasmids as the sole carriers of *mcr-1* without additional resistance genes [121,122,123,124], other investigations have documented variant IncX4 plasmids that co-harbored other resistance determinants. For instance, pMIMAEC11*mcr* and pMIMAEC91*mcr* from Brazil carried *bla*_TEM-1A_, *aph(6)-Id*, *aph(3″)-Ib*, and *qnrB19*, while another clinical isolate from São Paulo carried *aac(3)-iib*, *aph(3″)-Ib*, *aph(6)-Id*, *sul2*, *floR*, and *bla*_TEM-1_, reflecting a broader MDR phenotype [125,126,127,128].

IncX4 plasmids have been identified in a diverse range of bacterial species, most commonly *E. coli*, as well as in *S. enterica*, *K. pneumoniae*, and, less frequently, *P. aeruginosa* and *Enterobacter* spp. [85,110,113,129,130]. They have been isolated from clinical samples (blood, stool, urine, wound swabs, respiratory secretions), animal hosts (pigs, ducks, poultry), environmental sources (sewage, water), and foodborne samples (chicken carcasses, retail meat) across countries including China, Brazil, Thailand, Greece, Romania, Poland, Hungary, Belgium, and Germany (Table S2) [46,113,129].

Surveillance studies have shown that IncX4 plasmids can persist long-term in bacterial populations, even without colistin use [122,124,131]. However, policy interventions, such as the withdrawal of colistin from animal feed, have dramatically reduced their prevalence. In a pig farm in Sichuan, China, the detection of *mcr-1.1*-positive IncX4 plasmids dropped from 86.4% to 5.6% following a national colistin ban, emphasizing their sensitivity to antimicrobial use practices [124].

### 3.4. Other Less-Reported Plasmids

#### 3.4.1. Multi-Replicon Plasmids

Multireplicon plasmids carrying *mcr* genes exhibit remarkable diversity in size, replicon composition, and functional attributes. These plasmids range from compact 33 kb plasmids like pMIMAEC13-43 to expansive 350 kb plasmids like pEC15-MCR-50, with GC content averaging 48.0% in larger plasmids [102,106]. Replicon combinations are highly variable, encompassing IncHI2/IncFIB/IncN in pKP2509-MCR (317 kb) [132], IncFIA/IncHI1A/IncHI1B in pCP53-*mcr1_3* (231 kb) [133], and IncX1/IncFIA in pRW7-1 (235 kb) [134]. Transferability varies widely: conjugative plasmids like pKP14812-*MCR-1* transfer at frequencies of 1.18 × 10^−4^ [135], while others, such as pCP53-*mcr1_3*, lack conjugative machinery [133]. Plasmids with broad-host-range replicons (e.g., IncA/C, IncFIB) facilitate interspecies dissemination [136]. Smaller plasmids, such as pMIMAEC13-43 (33 kb), bypass reliance on common mobilization elements like IS*Apl1* through alternative mechanisms [106].

#### 3.4.2. IncF Plasmids

IncF plasmids are notable for their role in disseminating colistin resistance via the *mcr-1* gene alongside other multidrug resistance determinants. These plasmids vary in size, ranging from 60 kb (pMIMAEC08-85) to 131 kb (bovine mastitis isolate), and are transferable between bacterial strains, facilitating HGT [22,106,116]. A defining feature of IncF plasmids is the presence of IS, such as IS*Apl1*, which flank the *mcr-1* gene in some cases, aiding its mobilization [106,123]. Transposons are generally absent, simplifying their structure but not hindering their transferability [123,137]. These plasmids carry diverse resistance genes, including *aac(3)-IIc*, *bla*_CTX-M_, bla_TEM-1_, and *aph(3′)-Ib* [106,133,138]. The genetic environment of *mcr-1* in IncF plasmids often includes mobile elements like IS*Apl1*, though allelic variants such as *mcr-1.22* have been identified in poultry-associated strains, suggesting evolutionary adaptations under selective pressures [106,123]. Toxin-antitoxin systems, such as VapB/RelE2, contribute to plasmid stability and persistence [116].

#### 3.4.3. IncFIB Plasmids

IncFIB plasmids are broad-host-range vectors associated with high genetic diversity due to mobile genetic elements. Sizes range from 60 kb to over 150 kb [33], and they are either self-transmissible or conjugative, enabling the spread of resistance genes across bacterial species [91,139,140]. Insertion sequences, such as IS*Apl1* and IS*Kpn40*, are frequently observed near *mcr* genes, with IS*Kpn40* facilitating integration into transposons, including the Tn3-family elements [139,141]. These plasmids co-harbor resistance determinants for β-lactams (e.g., *bla*_CTX-M-55_, *bla*_CTX-M-9_), aminoglycosides, and carbapenems, amplifying their threat in clinical settings [139,140]. The *mcr-1.1* gene in plasmid pCAU16175_4 is flanked by IS*Apl1* but lacks transposons, indicating alternative mobilization mechanisms [123]. The genetic environments of *mcr* genes in IncFIB plasmids are often complex, featuring overlapping resistance cassettes and mobile elements that drive the dissemination of multidrug resistance [91,141].

#### 3.4.4. IncFII Plasmids

IncFII plasmids are large (76–150 kb) conjugative vectors prevalent in Enterobacteriaceae, particularly *E. coli* and *Salmonella* [142]. They exhibit high genetic variability due to mobile elements, such as IS26, ISKpn40, and ISApl1, which flank resistance genes and promote horizontal transfer [123,142]. These plasmids harbor extensive resistance profiles, including bla_OXA-48_, bla_VIM_, tet(M), and rmtB, which confer resistance to carbapenems, tetracyclines, and aminoglycosides [123,136]. Toxin–antitoxin systems like pemI/pemK enhance plasmid stability in hosts [137]. Conjugative transfer frequencies highlight their role in disseminating resistance across clinical and environmental settings [136].

#### 3.4.5. IncHI1 Plasmids

IncHI1 plasmids are large, broad-host-range vectors frequently identified in *K. pneumoniae* and *E. coli* from clinical and animal sources. Notably, these plasmids exhibit self-transferability via conjugation, facilitating cross-species gene dissemination [95]. The *mcr-1* gene is a hallmark of IncHI1 plasmids, often embedded within transposons such as Tn6330 or Tn6390, which are flanked by IS*Apl1* insertion sequences. These mobile elements enhance horizontal transfer, enabling regional dissemination in pig farms in Thailand [143].

IncHI1 plasmids are notable for their extensive resistance gene repertoires. For instance, pKP14812-MCR1 carries *aadA1*, *bla*_CMY-2_, and *tet*(M), while pKP16103-MCR1 harbors *aph(3″)-Ia* and *cmlA1* [95]. Co-localization of *mcr-1* with *bla*_CTX-M_ genes amplifies their role in multidrug resistance [143]. However, some IncHI1 plasmids lack detailed genetic context for *mcr-1*, as observed in *E. coli* isolates where neither transposons nor additional resistance genes were reported [144]. This variability underscores the adaptability of IncHI1 plasmids across ecological niches.

#### 3.4.6. Phage-like Plasmids

Phage-like plasmids, such as p0111, represent a unique mechanism for *mcr* gene dissemination. Isolated from *E. coli* in crab meat, p0111 resembles P1 bacteriophages and lacks transposons or insertion sequences [77,123]. Despite this simplicity, its phage-like structure enables *mcr-1*.*1* transfer via transduction, bypassing conjugation. Notably, p0111 carries no additional resistance genes, distinguishing it from MDR plasmids [123]. This phage–plasmid hybrid highlights an understudied route for resistance gene mobilization.

P1-like phage-plasmids are hybrid vectors identified in *Enterobacteriaceae*, carrying *mcr-1* and *tet*(X4) alongside β-lactamase genes. The *mcr-1* gene is embedded in an IS30-*mcr-1*-ORF-IS30 cluster, supported by Tn3-family transposons and class 1 integrons [145]. These plasmids also harbor phage tail fiber genes, suggesting dual transduction and conjugation mechanisms. Their ability to co-transfer tetracycline, macrolide, and β-lactam resistance genes underscores their threat in clinical and zoonotic contexts [146].

## 4. Chromosomal Integration of the *mcr* Gene

The chromosomal integration of *mcr* genes has been documented across diverse sources, including humans, animals, and environmental reservoirs (Table 2). Notably, *mcr-1*-positive strains with chromosomal integration have been isolated from healthy human carriers (6.3% prevalence) and colonized patients (2.7%) in surveillance studies, as well as from the fecal microbiota of healthy individuals in rural Vietnam (21/57 isolates), where the gene was embedded within the Tn6330 transposon [147,148]. Clinical human isolates include *K. pneumoniae* ST147 from a rectal sample of a hematologic patient in the Netherlands and *Aeromonas veronii* FC951 from a symptomatic patient’s stool in India, demonstrating chromosomal *mcr* integration [58,149]. Additionally, *Salmonella* Indiana S530, isolated from a diarrheal patient, harbored a chromosomally integrated (albeit non-functional) *mcr-1* gene [150]. Environmental and food sources are also implicated, with 1.4% of chromosomally encoded *mcr-1*-positive isolates originating from environmental samples and three *E. coli* strains detected in mutton and poultry meat [147,151]. Animal-derived isolates are extensive, spanning pigs, goats, poultry, and cattle, with chromosomal *mcr-1* frequently linked to Tn6330 or IS*Apl1*-mediated integration [122,152,153]. These findings underscore the gene’s adaptability across hosts and environments, emphasizing the need for integrated surveillance to track its dissemination.

Integrating the *mcr* gene family, particularly *mcr-1* and *mcr-3*, into bacterial chromosomes is a critical mechanism for colistin resistance, traditionally associated with plasmid-mediated horizontal transfer but increasingly recognized for its chromosomal stability [155]. This process is driven by mobile genetic elements (MGEs) such as the composite transposon Tn6330, which harbors the *mcr-1* gene flanked by IS*Apl1* insertion sequences (IS*Apl1*-*mcr-1*-*pap2*-IS*Apl1*), facilitating transposition into chromosomal loci via recombination [67,147]. In *E. coli* strain Q4552, a 51,089-bp MGE containing *mcr-1*.*1* integrated upstream of a tRNA-Met gene through phage integrase-mediated transposition, highlighting an alternative phage-driven pathway for chromosomal insertion, as described by Hamame et al. [137]. Additionally, IS*Apl1* elements target AT-rich intergenic regions, exemplified by *E. coli* HeN100, where *mcr-1* inserted between IS*Apl1* and a *PAP2*-like protein-coding gene, generating target site duplications (TSDs) characteristic of transposition, as observed by Peng et al. [153]. Recombination events further contribute to integration, where IS*Apl1* and *pap2* facilitated *mcr-1* insertion, though subsequent disruption by ISVsa5 inactivated the gene, illustrating context-dependent outcomes [157].

Structurally, chromosomally integrated *mcr* genes often reside within conserved genetic frameworks, such as the canonical IS*Apl1*-*mcr-1*-*pap2*-IS*Apl1* transposon, which preserves gene integrity while enabling mobility [147,148]. Some integration sites feature truncated phage-like sequences lacking lysogenic components, which may stabilize the gene by preventing excision [147]. Variability in insertion loci is evident, with *mcr-1* integrating into AT-rich regions, near toxin–antitoxin systems (e.g., *lysN/hicB*), or sporadically across strains, as seen in *K. pneumoniae* ST147, where multiple IS*Apl1* copies flank the gene, underscoring dynamic integration [58]. Stabilization is further enhanced by accessory systems, such as toxin–antitoxin and restriction–modification systems co-located within MGEs, as observed in *E. coli* Q4552, where these systems limit competing genetic elements to ensure persistence [137].

Chromosomal integration ensures stable vertical transmission of *mcr* genes, contrasting with plasmid-borne variants prone to loss without selective pressure, as demonstrated in pigeon-derived *E. coli* isolates where chromosomally integrated *mcr-1* remained non-transferable but stably inherited [154,156]. Despite this stability, residual mobilization potential persists; for example, IS*Apl1* elements in *E. coli* HeN100 may mediate *mcr-1* transfer to plasmids or other bacteria, perpetuating resistance [153]. Public health concerns arise from the persistence of chromosomal *mcr* genes, which evade plasmid-targeted interventions and endure without antibiotic selection, complicating resistance management [58,141]. Furthermore, integration outcomes vary widely: while some insertions stabilize resistance, others lead to gene inactivation, as seen with ISVsa5 disrupting *mcr-1* in *Salmonella* S530, reflecting fitness trade-offs [150]. This variability highlights the adaptability of *mcr* integration mechanisms and their context-dependent influence on bacterial fitness and the dissemination of resistance.

## 5. Insertion Sequences Driving *mcr* Mobilization

IS are critical mobile genetic elements facilitating the horizontal transfer of *mcr* genes, particularly *mcr-1*, across bacterial populations (Table 3). In IncI2 plasmids, *mcr-1* is frequently associated with IS elements that promote plasmid recombination, transposition, and mobilization, contributing to genetic plasticity and dissemination of colistin resistance [59,68,70]. IS*Apl1* is the most prevalent IS element in IncI2 plasmids, often flanking *mcr-1* in animal-derived isolates (e.g., pigs, poultry, wastewater) and forming the composite transposon Tn6330 (IS*Apl1*-*mcr-1*-*pap2*-IS*Apl1*), which enhances conjugative transfer across species and environments [69,84,158]. Other IS elements, including IS2, IS4, IS26, ISKpn26, and IS1294, further contribute to *mcr-1* mobilization in IncI2 plasmids [27,90,91,139,159]. Notably, IS*Apl1* is often absent in human-derived IncI2 plasmids, suggesting the existence of alternative mobilization mechanisms. In clinical *E. coli* isolates, *mcr-1* may reside in simpler genetic environments or rely on IS elements like IS2 or IS4 [33,70,90]. Some plasmids compensate for IS*Apl1* absence through structures such as the *nikB*-*mcr-1*-*pap2* cassette, observed in Vietnamese *E. coli* strains, indicating diverse mobilization pathways [156,160]. The absence of IS elements in some IncI2 plasmids may reduce transfer efficiency but enhance genetic stability, favoring persistence in clinical settings [81].

In contrast, *mcr*-carrying IncX4 plasmids exhibit a near-universal lack of IS elements, particularly IS*Apl1*, favoring a streamlined, stable structure. For instance, *mcr-1* in IncX4 plasmids is typically flanked by conserved DUF-domain genes (e.g., DUF2606 and DUF2726), suggesting vertical inheritance rather than IS-driven mobilization [59,125]. This IS-free architecture is globally consistent across human, animal, and environmental isolates [23,112,117,161]. However, reports of IncX4 plasmids harboring IS elements, like IS26, ISKpn26, and IS*Apl1*, have been reported [127,142,162].

IS*Apl1* is the most extensively documented IS element linked to *mcr-1* in IncHI2 plasmids. It is frequently identified upstream of *mcr-1* or flanking both ends to form the composite transposon Tn6330. The canonical IS*Apl1*–*mcr-1*–*pap2*–IS*Apl1* arrangement of Tn6330 has been observed across diverse bacterial hosts, including *E. coli*, *K. pneumoniae*, and *S. enterica*, underscoring its broad adaptive significance [95,163,164]. Variations in this structure, from complete to partial configurations, reflect dynamic stages of transposition or stabilization of *mcr-1* within plasmids. For instance, in *E. coli* Ec502 from Brazil and *Salmonella* Typhimurium 16–541, the absence of IS*Apl1* flanking *mcr-1* suggests genetic fixation after prior mobilization events [97,125]. Beyond IS*Apl1*, IS26 is another prominent IS in IncHI2 plasmids, often associated with multidrug resistance regions. IS26 facilitates transposon truncation (e.g., Tn2 upstream of *bla*_TEM-1_), plasmid fusion, and recombination under antimicrobial pressure [99,165]. Its proximity to *mcr-1* in plasmids like pLD91-1-MCR1 and coexistence with IS*Apl1* in pYUAHP105-*MCR* and pYUYZMC28-MCR underscores its role in shaping complex resistance gene environments [80,134]. Additional IS elements further diversify IncHI2 plasticity. IS5, IS2, IS1203, and IS1A are recurrently linked to resistance genes such as *fosA3* and *bla*_CTX-M-14_, emphasizing IS-mediated co-mobilization of diverse determinants [90,96,103].

**Table 3:** Summary of IS and Tn elements in different plasmids carrying the *mcr* gene.

Replicon Type	IS Elements	Tn Elements	Reference
**IncX4**			
	IS26	Tn2	[166]
	IS26	None	[167]
	ΔIS5	None	[115]
	ISEc69	None	[168]
	IS*Kpn26*	None	[142]
	IS26	None	[169]
	None	None	[116]
**IncHI2**			
	IS*Apl1*	None	[116]
	IS26	None	[150]
	IS26, IS*Apl1*	None	[68]
	None	None	[46]
**IncI2**			
	IS*Apl1*	Tn6330	[160]
	IS*Apl1*	None	[170]
	IS1, IS*Apl1*	None	[144]
	ISEcp1, IS*Apl1*	None	[80]
	None	None	[120,171],
**Hybrid Types**			
Hybrid (IncHI1A:IncHI1B)	IS*Apl1*	Tn6330	[83]
Hybrid (IncFIB/IncHI1B)	IS*Apl1*, IS*Ec33*	Tn6330-like	[135]
Hybrid (IncFIA(HI1), IncHI2)	IS*Apl1*	Tn6330-like	[135]
Hybrid (IncR/IncN)	IS903B, IS*Apl1*	Not specified	[138]

## 6. Transposon Dynamics in Resistance Spread

Transposons play a central role in the mobilization and spread of *mcr* genes across bacterial plasmids, although their roles vary significantly between plasmid replicons [172]. The composite transposon Tn6330 (IS*Apl1*-*mcr-1*-*pap2*-IS*Apl1*) is a key driver of *mcr-1* dissemination in both IncI2 and IncHI2 plasmids (Table 3). In IncI2 plasmids, Tn6330 facilitates HGT in agricultural and clinical settings, particularly in *E. coli* from poultry, pigs, and wastewater [95,141,143,173]. Similarly, IncHI2 plasmids frequently harbor Tn6330 within MDR regions, enabling the mobilization of *mcr-1* in pathogens such as *K. pneumoniae* and *Salmonella* [95,104,174]. Variants of Tn6330 with truncated IS*Apl1* elements are also observed, suggesting stabilization of *mcr-1* in plasmid backbones. Despite these similarities, IncI2 plasmids exhibit more significant variability, with some human clinical isolates lacking transposons entirely and instead carrying *mcr-1* in simpler cassettes (e.g., *nikB*-*mcr-1*-*pap2*), which may reduce mobility but enhance stability [70,125,175].

IncHI2 plasmids display broader transposon diversity beyond Tn6330, including Tn2, Tn21, Tn6010, and integron-associated elements (e.g., In0, In640), which co-mobilize resistance genes for beta-lactams (*bla*_CTX-M_), tetracyclines (*tet*(A)), and aminoglycosides (*aadA1*) [67,99,100]. For example, Tn21 in *E. coli* EC13049 links *mcr-1*.*1* to mercury resistance, enabling environmental co-selection [100]. IS26-mediated rearrangements and ΔTnAs2 structures also contribute to plasticity in *mcr-3*-carrying IncHI2 plasmids [103,165].

In contrast, IncX4 plasmids are defined by their lack of transposons flanking *mcr-1*. Global studies confirm that *mcr-1*-positive IncX4 plasmids, such as pKP15450-MCR1 and pTB602, rely on conjugation machinery rather than transposons for dissemination, maintaining minimalist, stable backbones [95,113].

The variability in transposon association across plasmids underscores distinct evolutionary strategies. While IncI2 and IncHI2 plasmids leverage transposons for adaptability and co-resistance, IncX4 plasmids prioritize structural stability and efficient conjugation. This divergence underscores the necessity for targeted surveillance in environments such as agriculture and wastewater systems, where transposon-driven resistance dissemination is prevalent [59,72,139].

## 7. Co-Selection of Antibiotic Resistance Determinants

Plasmids harboring the *mcr-1* gene often co-select resistance determinants across various antibiotic classes, further intensifying multidrug resistance in pathogens (Table 4). Beta-lactam resistance genes, such as *bla*_TEM-1_ and *bla*_CTX-M_ variants, are commonly shared among IncI2 and IncHI2 plasmids. These genes confer resistance to extended-spectrum cephalosporins and penicillins and are often physically linked to *mcr-1* within transposons or integrons, facilitating co-transfer [59,120,126]. Similarly, aminoglycoside resistance genes (*aadA*, *aph(3″)-Ib*, *aph(6)-Id*, *aac(3)-IV*) are prevalent in both plasmid types, compromising therapies reliant on gentamicin, tobramycin, and kanamycin. Tetracycline (*tet*(A)) and sulfonamide (*sul2*, *sul3*) resistance genes further overlap across IncI2 and IncHI2 plasmids, particularly in *E. coli* and *Salmonella* strains from livestock and clinical settings, amplifying risks of agricultural-to-human resistance transmission.

IncHI2 plasmids uniquely co-harbor broader resistance profiles, including phenicol (*floR*, *cmlA1*), quinolone (*qnrS1*/*2*, *oqxAB*), and fosfomycin (*fosA3*) resistance genes, often clustered within mobile genetic elements [69,142]. They also carry heavy metal (*mer, terABCDE*) and disinfectant (*qacE*/*L*) resistance operons, enhancing environmental persistence and co-selection under non-antibiotic pressures [98,103]. In contrast, IncI2 plasmids are frequently associated with macrolide (*mph(A*/*B*)) and phenicol (*catA1*) resistance, with *mph(A)* linked to clinical *Enterobacteriaceae* infections [72,90].

IncX4 plasmids exhibit a distinct pattern, typically carrying *mcr-1* alone in environmental and animal isolates [46,59]. However, clinical variants demonstrate recombination potential, co-integrating genes such as *bla*_NDM-1_, *bla*_KPC-1_, *aac(3)-IId*, *sul2*, and *floR* [110,125]. In Brazil, *mcr-1*-bearing IncX4 plasmids co-existed with chromosomal *qnrB19* and *bla*_TEM-1A_, highlighting risks of resistance convergence in human hosts [112]. Though minimalistic, their compatibility with other plasmids in multireplicon strains enables MDR amplification, as seen in *E. coli* isolates co-harboring *bla*_NDM-5_ and *tet*(A) [170].

Multireplicon plasmids accumulate resistance determinants from various replicon types, integrating *mcr-1* with last-resort resistance genes such as *bla*_NDM_ (carbapenems), *tmexCD1-toprJ1* (tigecycline), and *qnrS1* (quinolones) [106,135]. These plasmids, often isolated from clinical *Enterobacteriaceae*, exemplify pan-resistance convergence, rendering infections nearly untreatable [136,138].

The co-selection of *mcr-1* with diverse resistance genes across plasmid types underscores a critical public health challenge. Environmental and agricultural reservoirs perpetuate the dissemination of MDR plasmids, necessitating enhanced surveillance and stewardship to curb the spread of pan-resistant pathogens.

**Table 4:** Examples of resistance genes co-encoded with the *mcr* gene in plasmids harboring the *mcr* gene.

Antibiotic	Resistance Gene	Inc Group(s)	References
**Aminoglycosides**	*aac(3)-IIb*	IncX4, IncHI2A	[125]
	*aac(3)-IId*	IncX4	[126]
	*aac(6′)-Ib*	IncHI2	[95]
	*aadA1*	IncHI2, IncI2	[95,176]
	*aph(3″)-Ib*	IncX4, IncHI2A	[126]
**Beta-lactams**	*bla*_TEM1_	IncX4, IncHI2A, IncI2	[59]
	*bla*_CTX-M-14_	Hybrid (IncFII/IncFIA), IncHI2	[59,102]
	*bla*_NDM-1_	IncX4, IncI2	[76,110]
**Chloramphenicol**	*floR*	IncX4, IncHI2	[126]
	*cmlA1*	IncHI2, IncI2	[120]
**Sulfonamides**	*sul1*	IncHI2, IncI2	[95,176]
	*sul2*	IncX4, IncHI2A, IncI2	[125,126]
**Tetracyclines**	*Tet*(A)	IncHI2, IncI2, IncX4	[116,165],
	*tet*(M)	IncHI2, IncX1	[116,174]
**Quinolones**	*qnrS1*	IncHI2, IncI2	[78,90]
	*oqxAB*	IncHI2	[104]
**Macrolides**	*mph(A)*	IncHI2, IncX4	[59,126]

## 8. Conclusions

The global dissemination of plasmid-mediated *mcr* genes, particularly *mcr-1*, represents a critical challenge to public health, driven by the adaptability and mobility of resistance-bearing plasmids. Key plasmid families—IncI2, IncHI2, and IncX4—employ distinct evolutionary strategies to propagate colistin resistance. IncI2 plasmids thrive in clinical and livestock environments due to their stability and co-selection of multidrug resistance (MDR) determinants. IncHI2 plasmids leverage transposons and integrons to integrate *mcr* genes within complex resistance islands, facilitating the co-transfer of resistance to antibiotics, heavy metals, and disinfectants. In contrast, IncX4 plasmids achieve global spread through streamlined, conjugation-efficient architectures, often lacking mobile elements but maintaining persistence without selective pressure.

Integrating *mcr* genes into bacterial chromosomes via mobile genetic elements (MGEs) such as Tn6330 and phage-like systems further entrenches resistance, enabling stable vertical transmission and evading plasmid-targeted interventions. Insertion sequences (e.g., IS*Apl1*) and transposons drive HGT, while co-location with β-lactamase, aminoglycoside, and tetracycline resistance genes amplifies MDR phenotypes, rendering infections increasingly untreatable.

Environmental reservoirs, agricultural practices, and food chains serve as interconnected conduits for disseminating resistance, underscoring the One Health imperative. Overusing colistin in livestock, inadequate sanitation, and antibiotic stewardship perpetuate the cycle of resistance across ecosystems. Addressing this crisis demands coordinated action: stringent regulation of colistin in agriculture, enhanced surveillance of high-risk plasmid types, and international collaboration to curb cross-border transmission.

Ultimately, plasmids’ plasticity and ability to transcend ecological and taxonomic barriers underscore the urgent need for innovative strategies—ranging from phage therapy to CRISPR-based interventions—to disrupt the dissemination of resistance. Preserving colistin’s efficacy requires a holistic approach that bridges clinical, agricultural, and environmental sectors, safeguarding global health against the looming threat of pan-resistant infections.

## Figures and Tables

**Table 1:** Summary table of IncI2, IncX4, and IncHI2 plasmids retrieved from different sources and countries.

Replicon	Sources	Countries (Continents)	References
**IncI2**	Clinical, poultry, wastewater, pigs, meat	China (Asia), Pakistan (Asia), Brazil (South America), Europe (Greece, Poland, Netherlands), Africa (Nigeria, Tunisia)	[44,57,58,59]
**IncX4**	Clinical, poultry, pigs, meat, environment, water	China (Asia), Brazil (South America), Europe (Greece, Netherlands, Germany, Romania), Thailand (Asia), Africa (Egypt)	[46,54,58,59,60,61,62,63,64]
**IncHI2**	Clinical, animals (poultry, pigs), food, environment	China (Asia), Tunisia (Africa), Poland (Europe), Egypt (Africa), Brazil (South America)	[46,59,63,65,66]

**Table 2:** Some reported cases of *mcr-1* chromosomal integration: host species, integration mechanisms, and mobile genetic elements.

Host Source	Bacterial Species	Geographic Location	Chromosomal Integration Site/Mechanism	Associated Mobile Genetic Elements	Reference
Animal (goats)	*E. coli*	France	Integration via Tn6330 (composite transposon) at multiple chromosomal sites	Tn6330, IS*Apl1*	[122]
Animal (organs)	*E. coli*	China	Integration via Tn6330	Tn6330	[67]
Animal (pig stool)	*E. coli*	Avignon, France	Integration near tRNA-Met gene via phage integrase and IS30 transposases	IS30, phage integrase	[137]
Animal (pig)	*E. coli*	China	Triplication via Tn6330 in chromosomal regions	Tn6330, IS*Apl1*	[152]
Animal (pig)	*E. coli*	China	Transposition via IS*Apl1* into AT-rich regions with target site duplication	IS*Apl1*	[153]
Animal (pig), food (meat)	*E. coli*	Thailand	Chromosomal insertion via IS*Apl1*	IS*Apl1*	[141]
Animal (pigeons)	*E. coli*	China	Integration via Tn6330	Tn6330, IS*Apl1*	[154]
Food (poultry, mutton)	*E. coli*	India	Transposition via IS*Apl1* into AT-rich regions	IS*Apl1*	[151]
Human (clinical)	*A. veronii*	India	Chromosomal integration disrupted by IS*As18*	IS*As18*, IS*As19*, IS*As20*	[149]
Human (clinical)	*K. pneumoniae*	Nethelands	Integration via multiple IS*Apl1* elements	IS*Apl1*	[58]
Human (clinical)	*S. Indiana*	China	Recombination event involving IS*Apl1* and *pap2*, disrupted by IS*Vsa5*	IS*Apl1*, ISV*sa5*	[150]
Human (fecal)	*E. coli*	Vietnam	Integration via Tn6330 (IS*Apl1*-*mcr-1-pap2*-IS*Apl1*) at random chromosomal sites	Tn6330, IS*Apl1*	[148]
Human, animal, food	*E. coli*	China	Integration via Tn6330 (IS*Apl1*-*mcr-1*-*orf*-IS*Apl1* structure) into AT-rich regions	Tn6330, IS*Apl1*	[155]
Human, animal, food, water	*E. coli*	Vietnam	Integration via Tn6330 and IS*Apl1*	Tn6330, IS*Apl1*	[156]

## Data Availability

The data presented in this study are available within the article. Raw data supporting this study are available from the corresponding author upon reasonable request.

## Supplementary Materials

The following supporting information can be downloaded at: https://www.mdpi.com/article/10.3390/antibiotics14050506/s1, Table S1: Some reported prevalence of *mcr* in different samples and countries; Table S2: Characteristics of some reported plasmids encoding *mcr* gene.
