# Supplementary material for: One Health at Risk: Plasmid-Mediated Spread of mcr-1 Across Clinical, Agricultural, and Environmental Ecosystems
Source: Antibiotics (Basel). 2025 May 15;14(5):506. doi: 10.3390/antibiotics14050506 (PMC12108367; doi:10.3390/antibiotics14050506)
Supplement: Supplementary file 1 [file antibiotics-14-00506-s001.zip › antibiotics-3598466-supplementary.pdf]

Table S1: Some reported prevalence of mcr in different samples and countries

Origin	Country	Prevalence	Reference
Barbary macaques	Algeria	1/86 (1.2%)	[1]
Broiler chickens	Brazil	57.9% (62/107)	[2]
Chicken and the farm environment	Malaysia	10.8% (48/425) of isolates carried one or more colistin-resistance encoding genes	[3]
Livestock and food	Germany	402 isolates harboured the *mcr-1* gene among 505 colistin-resistant isolates	[4]
Livestocks	France	1.5 % (23 strains among 1450 *E. coli* strains tested)	[5]
Food animals	China	91% (182 were positive for *mcr-1* among 200 randomly selected colistin-resistant isolates)	[6]
Human clinical	Argentina	4.2% (8/192 clinical *E. coli* isolates)	[7]
Human clinical	Philippines	1.6% (2/123 antimicrobial-resistant isolates)	[8]
Human clinical	Nepal	0.6% (3/529 clinical samples)	[9]
Human clinical	China	2.8% (19/668 *E. coli* strains)	[10]
Human clinical	Pakistan	0.73% (4/545 *E. coli* strains)	[11]
Human clinical	Pakistan	66% (8/12 *K. pneumoniae* strains)	[12]
Human clinical	Turkey	5% (2/40 *E. coli* strains)	[13]
Crested ibis (*Nipponia nippon*)	China	8% (8/100 fecal samples)	[14]
Slaughterhouse wastewater (Dawen river)	China	20% (4/20 samples)	[15]
Wastwater	Germany	9.6% (8/83)	[16]
Seafood	South Africa	90% (134/144)	[17]
Aquatic Environments	Algeria	4% (18/445 GNB)	[18]
Sewage water and rivers	Spain	30 *mcr-1* strains among 195 Enterobacterales strains (8 rivers samples and 5 sewage samples)	[19]
Urban sewage	China	0.82% (3/366)	[20]
Fallow deer	Portugal	4/83 (4.8%)	[21]
Fecal carriage (children)	China	0.80% (21/2632 fecal samples)	[22]
Fecal samples (diarrheal patients)	China	3.9% (40/1026 fecal samples)	[23]
Fennec fox	Sudan	1.1% (1/88)	[24]

Healthy poultry (turkeys and broilers)	France	2% (47/2259)	[25]
Wild griffon vultures (Gyps fulvus)	Spain	1.9% (2/104 cloacal samples)	[26]
Gulls	Spain/Portugal	5 strains	[27]
Fecal carriage in healthy people	China	15% (774/5159 samples)	[28]
Fecal carriage in healthy people from hospitals	China	3.4% (370/10816 samples)	[29]
ICU patient samples	Egypt	1.25% (6/480 GNB)	[30]
Fresh vegatables (Leaf rape and spinach)	China	0.28% (2/712)	[31]
Livestock and poultry	China	5.1% (356/6991)	[32]
Fecal samples of migratory birds	China	2.1% (23/1060)	[33]
Migratory birds	Egypt	14.3% (20/140)	[34]
Multicenter clinical study	China	1.03% (300/29100 *E. coli* isolates)	[35]
Vegetables (Parsley, coriander, celery, lettuce, chard, beet)	Chile	0.42% (2/478)	[36]
Pediatric clinical samples	China	0.7% (5/672 clinical samples)	[37]
Père David's deer	China	69.1% (67/97)	[38]
Retail meat (chickens and pork)	Japan	8.1% (9/111)	[39]
Pigs, pig carcass and pork	Thailand, Lao PDR, Cambodia	4.2% (68/1619)	[40]
Food animals (pig and chicken)	China	0.8% (11/1505 anal swab samples)	[41]
Poultry production	China	High prevalence (no exact data)	[42]
Poultry (fecal samples)	Lebanon	6.8% (19/280)	[43]
Chicken and pig farms (fecal samples)	Peru	21.3% (42/197 *E. coli* isolates) in chickens and 12.5% (18/144 *E. coli* isolates) in pigs	[44]
Poultry farms	Vietnam	49.5% (93/188) in chicken farms and 25.1% (45/179) in farmers	[45]
Aquatic products	China	22.8% (28/123) in freshwater fish samples and 47.1% (16/34) in crocodile cecum samples.	[46]
Rabbits	China	8/55 (14.5%)	[47]
Raw chicken meat	Malaysia	52.1% (12/23)	[48]
Fecal carriage in healthy individuals	Singapore	7.3% (8/109)	[49]
Retail chicken	Egypt	19% (9/47)	[50]

Human (hospital)	Egypt	2 *E. coli* strains	[51]
Retail chicken	Netherlands	24.8% (53/214)	[52]
Retail chicken	Qatar	18 *E. coli* strains	[53]
Retail meats and eggs	China	13% (40/307)	[54]
Retail meats	Japan	21% (16/76)	[55]
Retail poultry meat	Czech Republic	46 *mcr-1* positive *E. coli* strains	[56]
Vegetables and food animals	South Korea	0.47% (7/1474)	[57]
Haihe river	China	100% (18/18 water samples)	[58]
Seals (fecal samples)	Scotland/ Liverpool	1.8% (1/54)	[59]
Pigs	Portugal	98% (98/100)	[60]
Healthy turkeys (fecal samples)	Serbia	0.5% (5/2167)	[61]
Swine gallbladder	Brazil	1/6 Salmonella Choleraesuis strains	[62]
Vampire bats	Peru	33% (11 strains)	[63]
Various fresh vegetables	China	0.5% (4/720)	[64]
Various vegetables (leafy greens)	Algeria	0.5% (2/400)	[65]
Livestock, wildlife, produre and other environmental sources	USA	0% (0/1000 STEC isolates)	[66]
White storks	Spain	0.9% (4/467)	[67]
Wild birds	Pakistan	11% (11/100)	[68]
Wild boars	Tunisia	2.9% (1/34)	[69]
Wild mammals	Portugal	1 strains isolated from fecal sample was studied	[70]
Wild rats	Bangladesh	2.6% (1/39)	[71]
Fecal carriage in healthy humans and primary care patients	Switzerland	0% (0/1144)	[72]
Fecal carriage from outpatients with diarrhea	China	4.2% (230/5354 Salmonella strains)	[73]
Human fecal carriage in hospitalized patients	China	7.5% (605/8022)	[74]
Fecal carriage among healthy children from rural communities	Bolivia	38.3% (129/337)	[75]
Fecal carriage among community children	Taiwan	2.4% (12/510)	[76]
Human Fecal carriage (hospital)	Hong Kong	2.08% (14/672)	[77]

Fecal carriage among health volunteers	China	7.8% (56/719)	[78]
Fecal carriage among adults	China	0.1% (1/685)	[79]
Fecal carriage in healthy children	China	35.8% (118/330)	[80]
Fecal carriage in pet owners	China	2.7% (8/299)	[81]
Fecal carriage among residents in institutions	Netherlands	0% (0/ 385)	[82]
Human fecal carriage during international travel	Germany/Netherlands	11.4% (15/132)	[83]
Human fecal carriage (hospital)	Singapoure	6% (12/201)	[84]
Fecal carriage in humans and livestock	Cambodge	1.9% (11/592)	[85]

References

1. Bachiri, T.; Lalaoui, R.; Bakour, S.; Allouache, M.; Belkebla, N.; Rolain, J.M.; Touati, A. First Report of the Plasmid-Mediated Colistin Resistance Gene Mcr-1 in Escherichia Coli ST405 Isolated from Wildlife in Bejaia, Algeria. *Microb. Drug Resist. Larchmt. N* **2018**, *24*, 890–895, doi:10.1089/mdr.2017.0026.
2. Barbieri, N.L.; Pimenta, R.L.; de Melo, D.A.; Nolan, L.K.; de Souza, M.M.S.; Logue, C.M. Mcr-1 Identified in Fecal Escherichia Coli and Avian Pathogenic E. Coli (APEC) From Brazil. *Front. Microbiol.* **2021**, *12*, doi:10.3389/fmicb.2021.659613.
3. Lemlem, M.; Aklilu, E.; Mohamed, M.; Kamaruzzaman, N.F.; Zakaria, Z.; Harun, A.; Devan, S.S.; Kamaruzaman, I.N.A.; Reduan, M.F.H.; Saravanan, M. Phenotypic and Genotypic Characterization of Colistin-Resistant Escherichia Coli with Mcr-4, Mcr-5, Mcr-6, and Mcr-9 Genes from Broiler Chicken and Farm Environment. *BMC Microbiol.* **2023**, *23*, 392, doi:10.1186/s12866-023-03118-y.
4. Irrgang, A.; Roschanski, N.; Tenhagen, B.-A.; Grobbel, M.; Skladnikiewicz-Ziemer, T.; Thomas, K.; Roesler, U.; Käsbohrer, A. Prevalence of Mcr-1 in E. Coli from Livestock and Food in Germany, 2010–2015. *PLoS ONE* **2016**, *11*, e0159863, doi:10.1371/journal.pone.0159863.
5. Perrin-Guyomard, A.; Bruneau, M.; Houée, P.; Deleurme, K.; Legrandois, P.; Poirier, C.; Soumet, C.; Sanders, P. Prevalence of Mcr-1 in Commensal Escherichia Coli from French Livestock, 2007 to 2014. *Euro Surveill. Bull. Eur. Sur Mal. Transm. Eur. Commun. Dis. Bull.* **2016**, *21*, doi:10.2807/1560-7917.ES.2016.21.6.30135.
6. Huang, J.; Deng, S.; Ren, J.; Tu, J.; Ye, M.; Wang, M. Characterization of a blaNDM-1-harboring Plasmid from a Salmonella Enterica Clinical Isolate in China. *Mol. Med. Rep.* **2017**, *16*, 1087–1092, doi:10.3892/mmr.2017.6733.
7. Martino, F.; Petroni, A.; Menocal, M.A.; Corso, A.; Melano, R.; Faccone, D. New Insights on Mcr-1-Harboring Plasmids from Human Clinical Escherichia Coli Isolates. *PloS One* **2024**, *19*, e0294820, doi:10.1371/journal.pone.0294820.
8. Velasco, J.M.S.; Valderama, M.T.G.; Margulieux, K.R.; Diones, P.C.S.; Reyes, A.M.B.; Leonardia, S.G.; Liao, C.P.; Chua, D.A.; Navarro, F.C.S.; Ruekit, S.; et al. First Report of the Mcr-1 Colistin Resistance Gene Identified in Two Escherichia Coli Isolates from

Clinical Samples, Philippines, 2018. *J. Glob. Antimicrob. Resist.* **2020**, *21*, 291–293, doi:10.1016/j.jgar.2019.12.018.
9. Karki, D.; Dhungel, B.; Bhandari, S.; Kunwar, A.; Joshi, P.R.; Shrestha, B.; Rijal, K.R.; Ghimire, P.; Banjara, M.R. Antibiotic Resistance and Detection of Plasmid Mediated Colistin Resistance Mcr-1 Gene among Escherichia Coli and Klebsiella Pneumoniae Isolated from Clinical Samples. *Gut Pathog.* **2021**, *13*, 45, doi:10.1186/s13099-021-00441-5.
10. Liu, Y.; Wang, Q.; Qi, T.; Zhang, M.; Chen, R.; Si, Z.; Li, J.; Jin, Y.; Xu, Q.; Li, P.; et al. Molecular Epidemiology of mcr-1-Positive Polymyxin B-Resistant Escherichia coli Producing Extended-Spectrum β-Lactamase (ESBL) in a Tertiary Hospital in Shandong, China. *Pol. J. Microbiol.* **2024**, *73*, 363–375, doi:10.33073/pjm-2024-032.
11. Bilal, H.; Rehman, T.U.; Khan, M.A.; Hameed, F.; Jian, Z.G.; Han, J.; Yang, X. Molecular Epidemiology of Mcr-1, Bla KPC-2, and Bla NDM-1 Harboring Clinically Isolated Escherichia Coli from Pakistan. *Infect. Drug Resist.* **2021**, *14*, 1467–1479, doi:10.2147/IDR.S302687.
12. Aslam, B.; Siddique, M.H.; Siddique, A.B.; Shafique, M.; Muzammil, S.; Khurshid, M.; Rasool, M.H.; Ahmad, M.; Chaudhry, T.H.; Amir, A.; et al. Distribution of Mcr-1 Harboring Hypervirulent Klebsiella Pneumoniae in Clinical Specimens and Lytic Activity of Bacteriophage KpnM Against Isolates. *Infect. Drug Resist.* **2022**, *15*, 5795–5811, doi:10.2147/IDR.S374503.
13. Özkaya, E.; Buruk, C.K.; Tosun, İ.; Toraman, B.; Kaklıkkaya, N.; Aydın, F. [Investigation of Plasmid Mediated mcr Colistin Resistance Gene in Clinical Enterobacterales Isolates]. *Mikrobiyol. Bul.* **2020**, *54*, 191–202, doi:10.5578/mb.69021.
14. Lu, J.; Yang, Y.; Wu, Y.; Liu, C.; Zeng, Y.; Lei, L.; Song, H.; Zhang, R. Escherichia Coli Carrying IncI2 Plasmid-Mediated Mcr-1 Genes in Crested Ibis (Nipponia Nippon). *J. Infect. Public Health* **2022**, *15*, 558–561, doi:10.1016/j.jiph.2022.03.016.
15. Wang, X.; Li, L.; Sun, F.; Wang, J.; Chang, W.; Chen, F.; Peng, J. Detection of Mcr-1-Positive Escherichia Coli in Slaughterhouse Wastewater Collected from Dawen River. *Vet. Med. Sci.* **2021**, *7*, 1587–1592, doi:10.1002/vms3.489.
16. Savin, M.; Bierbaum, G.; Hammerl, J.A.; Heinemann, C.; Parcina, M.; Sib, E.; Voigt, A.; Kreyenschmidt, J. ESKAPE Bacteria and Extended-Spectrum-β-Lactamase-Producing Escherichia Coli Isolated from Wastewater and Process Water from German Poultry Slaughterhouses. *Appl. Environ. Microbiol.* **2020**, *86*, e02748-19, doi:10.1128/AEM.02748-19.
17. Abioye, O.E.; Nontongana, N.; Osunla, C.A.; Okoh, A.I. Antibiotic Resistance and Virulence Genes Profiling of Vibrio Cholerae and Vibrio Mimicus Isolates from Some Seafood Collected at the Aquatic Environment and Wet Markets in Eastern Cape Province, South Africa. *PloS One* **2023**, *18*, e0290356, doi:10.1371/journal.pone.0290356.
18. Cherak, Z.; Loucif, L.; Bendjama, E.; Moussi, A.; Benbouza, A.; Grainat, N.; Rolain, J.-M. Dissemination of Carbapenemases and MCR-1 Producing Gram-Negative Bacteria in Aquatic Environments in Batna, Algeria. *Antibiot. Basel Switz.* **2022**, *11*, 1314, doi:10.3390/antibiotics11101314.
19. Ovejero, C.M.; Delgado-Blas, J.F.; Calero-Caceres, W.; Muniesa, M.; Gonzalez-Zorn, B. Spread of Mcr-1-Carrying Enterobacteriaceae in Sewage Water from Spain. *J. Antimicrob. Chemother.* **2017**, *72*, 1050–1053, doi:10.1093/jac/dkw533.
20. Zhang, Y.; Chen, J.; Yang, X.; Wu, Y.; Wang, Z.; Xu, Y.; Zhou, L.; Wang, J.; Jiao, X.; Sun, L. Emerging Mobile Colistin Resistance Gene Mcr-1 and Mcr-10 in Enterobacteriaceae Isolates From Urban Sewage in China. *Infect. Drug Resist.* **2025**, *18*, 1035–1048, doi:10.2147/IDR.S502067.
21. Torres, R.T.; Cunha, M.V.; Araujo, D.; Ferreira, H.; Fonseca, C.; Palmeira, J.D. Emergence of Colistin Resistance Genes (Mcr-1) in Escherichia Coli among Widely Distributed Wild Ungulates. *Environ. Pollut. Barking Essex 1987* **2021**, *291*, 118136, doi:10.1016/j.envpol.2021.118136.

22. Mai, J.; Liang, Z.; Xiong, Z.; Zhang, C.; Cai, H.; Yao, S.; Chen, X.; Liang, B.; Gao, F.; Huang, Z.; et al. Fecal Carriage and Molecular Epidemiology of Mcr-1-Harboring Escherichia Coli from Children in Southern China. *J. Infect. Public Health* **2023**, *16*, 1057–1063, doi:10.1016/j.jiph.2023.05.005.
23. Xie, J.; Liang, B.; Xu, X.; Yang, L.; Li, H.; Li, P.; Qiu, S.; Song, H. Identification of Mcr-1-Positive Multidrug-Resistant Escherichia Coli Isolates from Clinical Samples in Shanghai, China. *J. Glob. Antimicrob. Resist.* **2022**, *29*, 88–96, doi:10.1016/j.jgar.2022.02.008.
24. Feng, C.; Wen, P.; Xu, H.; Chi, X.; Li, S.; Yu, X.; Lin, X.; Wu, S.; Zheng, B. Emergence and Comparative Genomics Analysis of Extended-Spectrum-β-Lactamase-Producing Escherichia Coli Carrying Mcr-1 in Fennec Fox Imported from Sudan to China. *mSphere* **2019**, *4*, e00732-19, doi:10.1128/mSphere.00732-19.
25. Perrin-Guyomard, A.; Houée, P.; Lucas, P.; Felten, A.; Le Devendec, L.; Chauvin, C.; Kempf, I. Prevalence and Molecular Epidemiology of Mcr-Mediated Colistin-Resistance Escherichia Coli from Healthy Poultry in France after National Plan to Reduce Exposure to Colistin in Farm. *Front. Microbiol.* **2023**, *14*, 1254122, doi:10.3389/fmicb.2023.1254122.
26. Sevilla, E.; Marín, C.; Delgado-Blas, J.F.; González-Zorn, B.; Vega, S.; Kuijper, E.; Bolea, R.; Mainar-Jaime, R.C. Wild Griffon Vultures (Gyps Fulvus) Fed at Supplementary Feeding Stations: Potential Carriers of Pig Pathogens and Pig-Derived Antimicrobial Resistance? *Transbound. Emerg. Dis.* **2020**, *67*, 1295–1305, doi:10.1111/tbed.13470.
27. Ahlstrom, C.A.; Ramey, A.M.; Woksepp, H.; Bonnedahl, J. Early Emergence of Mcr-1-Positive Enterobacteriaceae in Gulls from Spain and Portugal. *Environ. Microbiol. Rep.* **2019**, *11*, 669–671, doi:10.1111/1758-2229.12779.
28. Shen, Y.; Zhou, H.; Xu, J.; Wang, Y.; Zhang, Q.; Walsh, T.R.; Shao, B.; Wu, C.; Hu, Y.; Yang, L.; et al. Anthropogenic and Environmental Factors Associated with High Incidence of Mcr-1 Carriage in Humans across China. *Nat. Microbiol.* **2018**, *3*, 1054–1062, doi:10.1038/s41564-018-0205-8.
29. Shen, Y.; Zhang, R.; Shao, D.; Yang, L.; Lu, J.; Liu, C.; Wang, X.; Jiang, J.; Wang, B.; Wu, C.; et al. Genomic Shift in Population Dynamics of Mcr-1-Positive Escherichia Coli in Human Carriage. *Genomics Proteomics Bioinformatics* **2022**, *20*, 1168–1179, doi:10.1016/j.gpb.2022.11.006.
30. Elbaradei, A.; Sayedahmed, M.S.; El-Sawaf, G.; Shawky, S.M. Screening of Mcr-1 among Gram-Negative Bacteria from Different Clinical Samples from ICU Patients in Alexandria, Egypt: One-Year Study. *Pol. J. Microbiol.* **2022**, *71*, 83–90, doi:10.33073/pjm-2022-011.
31. Liu, B.-T.; Song, F.-J. Emergence of Two Escherichia Coli Strains Co-Harboring Mcr-1 and Bla NDM in Fresh Vegetables from China. *Infect. Drug Resist.* **2019**, *12*, 2627–2635, doi:10.2147/IDR.S211746.
32. Lv, D.; Duan, R.; Fan, R.; Mu, H.; Liang, J.; Xiao, M.; He, Z.; Qin, S.; Yang, J.; Jing, H.; et al. blaNDM and Mcr-1 to Mcr-5 Gene Distribution Characteristics in Gut Specimens from Different Regions of China. *Antibiot. Basel Switz.* **2021**, *10*, 233, doi:10.3390/antibiotics10030233.
33. Zhang, W.; Lu, X.; Chen, S.; Liu, Y.; Peng, D.; Wang, Z.; Li, R. Molecular Epidemiology and Population Genomics of Tet(X4), blaNDM or Mcr-1 Positive Escherichia Coli from Migratory Birds in Southeast Coast of China. *Ecotoxicol. Environ. Saf.* **2022**, *244*, 114032, doi:10.1016/j.ecoenv.2022.114032.
34. Ahmed, Z.S.; Elshafiee, E.A.; Khalefa, H.S.; Kadry, M.; Hamza, D.A. Evidence of Colistin Resistance Genes (Mcr-1 and Mcr-2) in Wild Birds and Its Public Health Implication in Egypt. *Antimicrob. Resist. Infect. Control* **2019**, *8*, 197, doi:10.1186/s13756-019-0657-5.
35. Jiang, Y.; Zhang, Y.; Lu, J.; Wang, Q.; Cui, Y.; Wang, Y.; Quan, J.; Zhao, D.; Du, X.; Liu, H.; et al. Clinical Relevance and Plasmid Dynamics of Mcr-1-Positive Escherichia Coli

in China: A Multicentre Case-Control and Molecular Epidemiological Study. *Lancet Microbe* **2020**, *1*, e24–e33, doi:10.1016/S2666-5247(20)30001-X.
36. Díaz-Gavidia, C.; Barría, C.; Rivas, L.; García, P.; Alvarez, F.P.; González-Rocha, G.; Opazo-Capurro, A.; Araos, R.; Munita, J.M.; Cortes, S.; et al. Isolation of Ciprofloxacin and Ceftazidime-Resistant Enterobacterales From Vegetables and River Water Is Strongly Associated With the Season and the Sample Type. *Front. Microbiol.* **2021**, *12*, 604567, doi:10.3389/fmicb.2021.604567.
37. Fan, Z.; Feng, Y.; Xu, W.; Feng, J.; Yan, C.; Fu, T.; Zhao, H.; Cui, J.; Gan, L.; Liu, S.; et al. Rapid Detection of Multi-Resistance Strains Carrying Mcr-1 Gene Using Recombinase-Aided Amplification Directly on Clinical Samples. *Front. Microbiol.* **2022**, *13*, 852488, doi:10.3389/fmicb.2022.852488.
38. Lu, X.; Xiao, X.; Liu, Y.; Huang, S.; Li, R.; Wang, Z. Widespread Prevalence of Plasmid-Mediated Colistin Resistance Gene Mcr-1 in Escherichia Coli from Père David's Deer in China. *mSphere* **2020**, *5*, e01221-20, doi:10.1128/mSphere.01221-20.
39. Nishino, Y.; Shimojima, Y.; Suzuki, Y.; Ida, M.; Fukui, R.; Kuroda, S.; Hirai, A.; Sadamasu, K. Detection of the Mcr-1 Gene in Colistin-Resistant Escherichia Coli from Retail Meat in Japan. *Microbiol. Immunol.* **2017**, *61*, 554–557, doi:10.1111/1348-0421.12549.
40. Pungpian, C.; Lee, S.; Trongjit, S.; Sinwat, N.; Angkititrakul, S.; Prathan, R.; Srisanga, S.; Chuanchuen, R. Colistin Resistance and Plasmid-Mediated Mcr Genes in Escherichia Coli and Salmonella Isolated from Pigs, Pig Carcass and Pork in Thailand, Lao PDR and Cambodia Border Provinces. *J. Vet. Sci.* **2021**, *22*, e68, doi:10.4142/jvs.2021.22.e68.
41. Ma, J.; Zhou, W.; Wu, J.; Liu, X.; Lin, J.; Ji, X.; Lin, H.; Wang, J.; Jiang, H.; Zhou, Q.; et al. Large-Scale Studies on Antimicrobial Resistance and Molecular Characterization of Escherichia Coli from Food Animals in Developed Areas of Eastern China. *Microbiol. Spectr.* **2022**, *10*, e0201522, doi:10.1128/spectrum.02015-22.
42. Wang, Y.; Zhang, R.; Li, J.; Wu, Z.; Yin, W.; Schwarz, S.; Tyrrell, J.M.; Zheng, Y.; Wang, S.; Shen, Z.; et al. Comprehensive Resistome Analysis Reveals the Prevalence of NDM and MCR-1 in Chinese Poultry Production. *Nat. Microbiol.* **2017**, *2*, 16260, doi:10.1038/nmicrobiol.2016.260.
43. Mikhayel, M.; Leclercq, S.O.; Sarkis, D.K.; Doublet, B. Occurrence of the Colistin Resistance Gene Mcr-1 and Additional Antibiotic Resistance Genes in ESBL/AmpC-Producing Escherichia Coli from Poultry in Lebanon: A Nationwide Survey. *Microbiol. Spectr.* **2021**, *9*, e0002521, doi:10.1128/Spectrum.00025-21.
44. Carhuaricra, D.; Duran Gonzales, C.G.; Rodríguez Cueva, C.L.; Ignacion León, Y.; Silvestre Espejo, T.; Marcelo Monge, G.; Rosadio Alcántara, R.H.; Lincopan, N.; Espinoza, L.L.; Maturrano Hernández, L. Occurrence and Genomic Characterization of Mcr-1-Harboring Escherichia Coli Isolates from Chicken and Pig Farms in Lima, Peru. *Antibiot. Basel Switz.* **2022**, *11*, 1781, doi:10.3390/antibiotics11121781.
45. Trung, N.V.; Matamoros, S.; Carrique-Mas, J.J.; Nghia, N.H.; Nhung, N.T.; Chieu, T.T.B.; Mai, H.H.; van Rooijen, W.; Campbell, J.; Wagenaar, J.A.; et al. Zoonotic Transmission of Mcr-1 Colistin Resistance Gene from Small-Scale Poultry Farms, Vietnam. *Emerg. Infect. Dis.* **2017**, *23*, 529–532, doi:10.3201/eid2303.161553.
46. Wang, C.-Z.; Li, X.-P.; Zhang, Y.-J.; Zhong, W.-C.; Liu, Y.-H.; Liao, X.-P.; Sun, J.; Zhou, Y.-F. Molecular Characteristic of Mcr-1 Gene in Escherichia Coli from Aquatic Products in Guangdong, China. *J. Glob. Antimicrob. Resist.* **2024**, *36*, 36–40, doi:10.1016/j.jgar.2023.11.010.
47. Wang, X.; Zhai, Z.; Zhao, X.; Zhang, H.; Jiang, H.; Wang, X.; Wang, H.; Chang, W. Occurrence and Characteristics of Escherichia Coli Mcr-1-like in Rabbits in Shandong, China. *Vet. Med. Sci.* **2021**, *7*, 219–225, doi:10.1002/vms3.340.
48. Aklilu, E.; Raman, K. MCR-1 Gene Encoded Colistin-Resistant Escherichia Coli in Raw Chicken Meat and Bean Sprouts in Malaysia. *Int. J. Microbiol.* **2020**, *2020*, 8853582, doi:10.1155/2020/8853582.

49. Ding, Y.; Saw, W.-Y.; Tan, L.W.L.; Moong, D.K.N.; Nagarajan, N.; Teo, Y.Y.; Seedorf, H. Extended-Spectrum β-Lactamase-Producing and Mcr-1-Positive Escherichia Coli from the Gut Microbiota of Healthy Singaporeans. *Appl. Environ. Microbiol.* **2021**, *87*, e0048821, doi:10.1128/AEM.00488-21.
50. Sadek, M.; Ortiz de la Rosa, J.M.; Abdelfattah Maky, M.; Korashe Dandrawy, M.; Nordmann, P.; Poirel, L. Genomic Features of MCR-1 and Extended-Spectrum β-Lactamase-Producing Enterobacterales from Retail Raw Chicken in Egypt. *Microorganisms* **2021**, *9*, 195, doi:10.3390/microorganisms9010195.
51. Soliman, A.M.; Ramadan, H.; Yu, L.; Hisatsune, J.; Sugai, M.; Elnahriry, S.S.; Nariya, H.; El-Domany, R.A.; Shimamoto, T.; Jackson, C.R.; et al. Complete Genome Sequences of Two Escherichia Coli Clinical Isolates from Egypt Carrying Mcr-1 on IncP and IncX4 Plasmids. *Front. Microbiol.* **2022**, *13*, 989045, doi:10.3389/fmicb.2022.989045.
52. Schrauwen, E.J.A.; Huizinga, P.; van Spreuwel, N.; Verhulst, C.; Kluytmans-van den Bergh, M.F.Q.; Kluytmans, J.A.J.W. High Prevalence of the Mcr-1 Gene in Retail Chicken Meat in the Netherlands in 2015. *Antimicrob. Resist. Infect. Control* **2017**, *6*, 83, doi:10.1186/s13756-017-0242-8.
53. Al Mana, H.; Johar, A.A.; Kassem, I.I.; Eltai, N.O. Transmissibility and Persistence of the Plasmid-Borne Mobile Colistin Resistance Gene, Mcr-1, Harbored in Poultry-Associated E. Coli. *Antibiot. Basel Switz.* **2022**, *11*, 774, doi:10.3390/antibiotics11060774.
54. Li, C.; Gu, X.; Zhang, L.; Liu, Y.; Li, Y.; Zou, M.; Liu, B. The Occurrence and Genomic Characteristics of Mcr-1-Harboring Salmonella from Retail Meats and Eggs in Qingdao, China. *Foods* **2022**, *11*, 3854, doi:10.3390/foods11233854.
55. Odoi, J.O.; Takayanagi, S.; Sugiyama, M.; Usui, M.; Tamura, Y.; Asai, T. Prevalence of Colistin-Resistant Bacteria among Retail Meats in Japan. *Food Saf. Tokyo Jpn.* **2021**, *9*, 48–56, doi:10.14252/foodsafetyfscj.D-21-00002.
56. Kubelová, M.; Koláčková, I.; Gelbíčová, T.; Florianová, M.; Kalová, A.; Karpíšková, R. Virulence Properties of Mcr-1-Positive Escherichia Coli Isolated from Retail Poultry Meat. *Microorganisms* **2021**, *9*, 308, doi:10.3390/microorganisms9020308.
57. Oh, S.-S.; Song, J.; Kim, J.; Shin, J. Increasing Prevalence of Multidrug-Resistant Mcr-1-Positive Escherichia Coli Isolates from Fresh Vegetables and Healthy Food Animals in South Korea. *Int. J. Infect. Dis. IJID Off. Publ. Int. Soc. Infect. Dis.* **2020**, *92*, 53–55, doi:10.1016/j.ijid.2019.12.025.
58. Yang, D.; Qiu, Z.; Shen, Z.; Zhao, H.; Jin, M.; Li, H.; Liu, W.; Li, J.-W. The Occurrence of the Colistin Resistance Gene Mcr-1 in the Haihe River (China). *Int. J. Environ. Res. Public. Health* **2017**, *14*, 576, doi:10.3390/ijerph14060576.
59. Watson, E.; Hamilton, S.; Silva, N.; Moss, S.; Watkins, C.; Baily, J.; Forster, T.; Hall, A.J.; Dagleish, M.P. Variations in Antimicrobial Resistance Genes Present in the Rectal Faeces of Seals in Scottish and Liverpool Bay Coastal Waters. *Environ. Pollut. Barking Essex 1987* **2024**, *349*, 123936, doi:10.1016/j.envpol.2024.123936.
60. Kieffer, N.; Aires-de-Sousa, M.; Nordmann, P.; Poirel, L. High Rate of MCR-1-Producing Escherichia Coli and Klebsiella Pneumoniae among Pigs, Portugal. *Emerg. Infect. Dis.* **2017**, *23*, 2023–2029, doi:10.3201/eid2312.170883.
61. Mišić, D.; Kiskaroly, F.; Szostak, M.P.; Cabal, A.; Ruppitsch, W.; Bernreiter-Hofer, T.; Milovanovic, V.; Feßler, A.T.; Allerberger, F.; Spergser, J.; et al. The First Report of Mcr-1-Carrying Escherichia Coli Originating from Animals in Serbia. *Antibiot. Basel Switz.* **2021**, *10*, 1063, doi:10.3390/antibiotics10091063.
62. Vilela, F.P.; Rodrigues, D.D.P.; Ferreira, J.C.; Darini, A.L. da C.; Allard, M.W.; Falcão, J.P. Genomic Characterization of Salmonella Enterica Serovar Choleraesuis from Brazil Reveals a Swine Gallbladder Isolate Harboring Colistin Resistance Gene Mcr-1.1. *Braz. J. Microbiol. Publ. Braz. Soc. Microbiol.* **2022**, *53*, 1799–1806, doi:10.1007/s42770-022-00812-3.
63. Benavides, J.A.; Godreuil, S.; Opazo-Capurro, A.; Mahamat, O.O.; Falcon, N.; Oravcova, K.; Streicker, D.G.; Shiva, C. Long-Term Maintenance of Multidrug-Resistant

Escherichia Coli Carried by Vampire Bats and Shared with Livestock in Peru. *Sci. Total Environ.* **2022**, *810*, 152045, doi:10.1016/j.scitotenv.2021.152045.
64. Li, C.-A.; Guo, C.-H.; Yang, T.-Y.; Li, F.-Y.; Song, F.-J.; Liu, B.-T. Whole-Genome Analysis of blaNDM-Bearing Proteus Mirabilis Isolates and Mcr-1-Positive Escherichia Coli Isolates Carrying blaNDM from the Same Fresh Vegetables in China. *Foods Basel Switz.* **2023**, *12*, 492, doi:10.3390/foods12030492.
65. Chelaghma, W.; Loucif, L.; Bendjama, E.; Cherak, Z.; Bendahou, M.; Rolain, J.-M. Occurrence of Extended Spectrum Cephalosporin-, Carbapenem- and Colistin-Resistant Gram-Negative Bacteria in Fresh Vegetables, an Increasing Human Health Concern in Algeria. *Antibiot. Basel Switz.* **2022**, *11*, 988, doi:10.3390/antibiotics11080988.
66. Mavrici, D.; Yambao, J.C.; Lee, B.G.; Quiñones, B.; He, X. Screening for the Presence of Mcr-1/Mcr-2 Genes in Shiga Toxin-Producing Escherichia Coli Recovered from a Major Produce-Production Region in California. *PloS One* **2017**, *12*, e0187827, doi:10.1371/journal.pone.0187827.
67. Höfle, U.; Jose Gonzalez-Lopez, J.; Camacho, M.C.; Solà-Ginés, M.; Moreno-Mingorance, A.; Manuel Hernández, J.; De La Puente, J.; Pineda-Pampliega, J.; Aguirre, J.I.; Torres-Medina, F.; et al. Foraging at Solid Urban Waste Disposal Sites as Risk Factor for Cephalosporin and Colistin Resistant Escherichia Coli Carriage in White Storks (Ciconia Ciconia). *Front. Microbiol.* **2020**, *11*, 1397, doi:10.3389/fmicb.2020.01397.
68. Munir, A.; Lu, X.; Humak, F.; Kurekci, C.; Mahmood, S.; Gul, S.; Wang, Z.; Mohsin, M.; Li, R. Emergence of Plasmid-Mediated Fosfomycin Resistance among Escherichia Coli Harboring fosA4, Tet(X4), and Mcr-1 Genes in Wild Birds. *mSystems®* **2025**, doi:10.1128/msystems.01673-24.
69. Selmi, R.; Tayh, G.; Srairi, S.; Mamlouk, A.; Ben Chehida, F.; Lahmar, S.; Bouslama, M.; Daaloul-Jedidi, M.; Messadi, L. Prevalence, Risk Factors and Emergence of Extended-Spectrum β-Lactamase Producing-, Carbapenem- and Colistin-Resistant Enterobacterales Isolated from Wild Boar (Sus Scrofa) in Tunisia. *Microb. Pathog.* **2022**, *163*, 105385, doi:10.1016/j.micpath.2021.105385.
70. Dantas Palmeira, J.; V Cunha, M.; Ferreira, H.; Fonseca, C.; Tinoco Torres, R. Worldwide Disseminated IncX4 Plasmid Carrying Mcr-1 Arrives to Wild Mammal in Portugal. *Microbiol. Spectr.* **2022**, *10*, e0124522, doi:10.1128/spectrum.01245-22.
71. Ali, M.W.; Karmakar, S.; Utsho, K.S.; Kabir, A.; Arif, M.; Islam, M.S.; Rahman, M.T.; Hassan, J. First Detection and Characterization of Mcr-1 Colistin Resistant E. Coli from Wild Rat in Bangladesh. *PloS One* **2024**, *19*, e0296109, doi:10.1371/journal.pone.0296109.
72. Zurfluh, K.; Stephan, R.; Widmer, A.; Poirel, L.; Nordmann, P.; Nüesch, H.-J.; Hächler, H.; Nüesch-Inderbinen, M. Screening for Fecal Carriage of MCR-Producing Enterobacteriaceae in Healthy Humans and Primary Care Patients. *Antimicrob. Resist. Infect. Control* **2017**, *6*, 28, doi:10.1186/s13756-017-0186-z.
73. Sun, R.-Y.; Fang, L.-X.; Ke, B.-X.; Sun, J.; Wu, Z.-W.; Feng, Y.-J.; Liu, Y.-H.; Ke, C.-W.; Liao, X.-P. Carriage and Transmission of Mcr-1 in Salmonella Typhimurium and Its Monophasic 1,4,[5],12:I:- Variants from Diarrheal Outpatients: A 10-Year Genomic Epidemiology in Guangdong, Southern China. *Microbiol. Spectr.* **2023**, *11*, e0311922, doi:10.1128/spectrum.03119-22.
74. Zhong, L.-L.; Phan, H.T.T.; Shen, C.; Vihta, K.-D.; Sheppard, A.E.; Huang, X.; Zeng, K.-J.; Li, H.-Y.; Zhang, X.-F.; Patil, S.; et al. High Rates of Human Fecal Carriage of Mcr-1-Positive Multidrug-Resistant Enterobacteriaceae Emerge in China in Association With Successful Plasmid Families. *Clin. Infect. Dis. Off. Publ. Infect. Dis. Soc. Am.* **2018**, *66*, 676–685, doi:10.1093/cid/cix885.
75. Giani, T.; Sennati, S.; Antonelli, A.; Di Pilato, V.; di Maggio, T.; Mantella, A.; Niccolai, C.; Spinicci, M.; Monasterio, J.; Castellanos, P.; et al. High Prevalence of Carriage of Mcr-1-Positive Enteric Bacteria among Healthy Children from Rural Communities in the Chaco Region, Bolivia, September to October 2016. *Euro Surveill. Bull. Eur. Sur Mal.*

Transm. Eur. Commun. Dis. Bull. **2018**, *23*, 1800115, doi:10.2807/1560-7917.ES.2018.23.45.1800115.
76. Wu, P.-C.; Cheng, M.-F.; Chen, W.-L.; Hung, W.-Y.; Wang, J.-L.; Hung, C.-H. Risk Factors and Prevalence of Mcr-1-Positive Escherichia Coli in Fecal Carriages Among Community Children in Southern Taiwan. *Front. Microbiol.* **2021**, *12*, 748525, doi:10.3389/fmicb.2021.748525.
77. Chan, W.-S.; Au, C.-H.; Ho, D.N.; Chan, T.-L.; Ma, E.S.-K.; Tang, B.S.-F. Prospective Study on Human Fecal Carriage of Enterobacteriaceae Possessing Mcr-1 and Mcr-2 Genes in a Regional Hospital in Hong Kong. *BMC Infect. Dis.* **2018**, *18*, 81, doi:10.1186/s12879-018-2987-y.
78. Lv, Z.; Shen, Y.; Liu, W.; Ye, H.; Liu, D.; Liu, J.; Fu, Y.; Peng, C.; Chen, K.; Deng, X.; et al. Prevalence and Risk Factors of Mcr-1-Positive Volunteers after Colistin Banning as Animal Growth Promoter in China: A Community-Based Case-Control Study. *Clin. Microbiol. Infect. Off. Publ. Eur. Soc. Clin. Microbiol. Infect. Dis.* **2022**, *28*, 267–272, doi:10.1016/j.cmi.2021.06.033.
79. Li, Y.; Ma, L.; Ding, X.; Zhang, R. Fecal Carriage and Genetic Characteristics of Carbapenem-Resistant Enterobacterales among Adults from Four Provinces of China. *Front. Epidemiol.* **2023**, *3*, 1304324, doi:10.3389/fepid.2023.1304324.
80. Liu, X.; Li, X.; Yang, A.-W.; Tang, B.; Jian, Z.-J.; Zhong, Y.-M.; Li, H.-L.; Li, Y.-M.; Yan, Q.; Liang, X.-H.; et al. Community Fecal Carriage and Molecular Epidemiology of Extended-Spectrum β-Lactamase- and Carbapenemase-Producing Escherichia Coli from Healthy Children in the Central South China. *Infect. Drug Resist.* **2022**, *15*, 1601–1611, doi:10.2147/IDR.S357090.
81. Lei, L.; Wang, Y.; He, J.; Cai, C.; Liu, Q.; Yang, D.; Zou, Z.; Shi, L.; Jia, J.; Wang, Y.; et al. Prevalence and Risk Analysis of Mobile Colistin Resistance and Extended-Spectrum β-Lactamase Genes Carriage in Pet Dogs and Their Owners: A Population Based Cross-Sectional Study. *Emerg. Microbes Infect.* **2021**, *10*, 242–251, doi:10.1080/22221751.2021.1882884.
82. van Dulm, E.; Klok, S.; Boyd, A.; Joore, I.K.; Prins, M.; van Dam, A.P.; Tramper-Stranders, G.A.; van Duijnhoven, Y.T.H.P. Nasal Carriage of Methicillin-Resistant Staphylococcus Aureus (MRSA) among Undocumented Migrants and Uninsured Legal Residents in Amsterdam, the Netherlands: A Cross-Sectional Study. *Antimicrob. Resist. Infect. Control* **2020**, *9*, 118, doi:10.1186/s13756-020-00785-8.
83. Schaumburg, F.; Sertic, S.M.; Correa-Martinez, C.; Mellmann, A.; Köck, R.; Becker, K. Acquisition and Colonization Dynamics of Antimicrobial-Resistant Bacteria during International Travel: A Prospective Cohort Study. *Clin. Microbiol. Infect. Off. Publ. Eur. Soc. Clin. Microbiol. Infect. Dis.* **2019**, *25*, 1287.e1-1287.e7, doi:10.1016/j.cmi.2019.03.002.
84. La, M.-V.; Lee, B.; Hong, B.Z.M.; Yah, J.Y.; Koo, S.-H.; Jiang, B.; Ng, L.S.Y.; Tan, T.-Y. Prevalence and Antibiotic Susceptibility of Colistin-Resistance Gene (*Mcr-1*) Positive *Enterobacteriaceae* in Stool Specimens of Patients Attending a Tertiary Care Hospital in Singapore. *Int. J. Infect. Dis.* **2019**, *85*, 124–126, doi:10.1016/j.ijid.2019.05.029.
85. Atterby, C.; Osbjer, K.; Tepper, V.; Rajala, E.; Hernandez, J.; Seng, S.; Holl, D.; Bonnedahl, J.; Börjesson, S.; Magnusson, U.; et al. Carriage of Carbapenemase- and Extended-Spectrum Cephalosporinase-Producing Escherichia Coli and Klebsiella Pneumoniae in Humans and Livestock in Rural Cambodia; Gender and Age Differences and Detection of blaOXA-48 in Humans. *Zoonoses Public Health* **2019**, *66*, 603–617, doi:10.1111/zph.12612.

Table S2: Characteristics of some reported plasmids encoding *mcr* gene

Replicon Type	*mcr variant*	*Species*	Source	Country	IS	Tn	Other antibiotic resistance genes (plasmid)	Reference
IncX4	*mcr-1*	*Salmonella serotype Goldcoast*	Duck feces	China	None	None	Not specified	[1]
IncHI2	*mcr-1*	*Salmonella serotype Ngor*	Duck feces	China	Not specified	None	*floR, tet(A),* bla_{TEM-1}, bla_{OXA-1}	[1]
IncX4	*mcr-1.1*	*E. coli*	Pediatric patient	Greece	None	None	None	[10]
IncX4	*mcr-1.1*	*Enterobacteriaceae*	Clinical	Korea	None	None	None	[11]
IncHI2	*mcr-1*	*S. enterica*	Clinical	Poland	IS1	Not specified	bla_{TEM-1B}, *mef(B), aadA1, qacL, dfrA12, aadA2, cmlA1, sul3, tet(M)*	[12]
IncX4	*mcr-1*	*E. coli*	Children with diarrhoea	China	IS26	None	bla_{TEM-1}, bla_{OXA-1}, *qnrS1, catB3*	[13]
IncI2	*mcr-1*	*E. coli*	Children with diarrhoea	China	None	None	bla_{TEM-1}, bla_{OXA-1}, *qnrS2*	[13]
Hybrid (IncX4, IncFIB, etc)	*mcr-1, mcr-3*	*E. coli and K. pneumoniae*	Clinical (pediatric patients)	China	Not specified	Not specified	ESBL, MBL, aminoglycosides, fluoroquinolones, tetracycline	[14]
IncI2	*mcr-1.1*	*E. coli*	Clinical	China	None	None	None	[15]
IncX4	*mcr-1.1*	*E. coli*	Clinical	Brazil	ΔIS5	None	Unspecified	[16]
IncX4	*mcr-1*	*Salmonella Typhimurium*	Pork	China	IS26	None	None	[17]

IncI2	*mcr-1*	*E. coli*	Duck farms	China	IS*Apl1*	None	*aac, aadA, bla$_{CTX}$, bla$_{TEM}$, sul1, sul2, tet, qnrS*	[18]
IncHI2	*mcr-1*	*S. enterica*	Foodborne(human and pig)	China	IS26, IS*Apl1*	None	*bla$_{CTX-M-14}$, aac(3)-IV, aph(4)-Ia, aadA1, aadA2b, oqxAB, cmlA1, floR, sul1, sul2, sul3, dfrA12*	[19]
IncI2	*mcr-1*	*S. enterica*	Foodborne(human and pig)	China	Not specified	None	None	[19]
IncI2	*mcr-1.1*	*E. coli*	Dairy farm wastewater sample	Pakistan	None	None	*bla$_{TEM-1B}$, tetB, catA1, mdfA, dfrA17, aadA5, aph(3'')-Ib, aph(6)-Id, sul2*	[2]
IncI2	*mcr-1*	*E. coli*	Chicken feces	China	None	None	None	[20]
IncHI2	*mcr-3.1*	*E. coli*	Porcine sources	China	IS26, IS6100	ΔTnAs2	Beta-lactams, aminoglycosides, sulfonamides	[21]
IncP1	*mcr-3.1/3.5*	*E. coli*	Porcine sources	China	IS4321, ISKpn40	None	Tetracycline, florfenicol, β-lactams	[21]
P1-like	*mcr-1*	*Enterobacteriaceae spp.*	Animals/clinical	China	IS30	Tn3 family	tet(X4), *bla*$_{CTX-M-55}$, *bla*$_{TEM-95}$, tetracycline, chloramphenicol, macrolides	[22]
IncX4	*mcr-1*	*E. coli*	Clinical	-	IS*Apl1*	Not specified	Beta-lactams, fluoroquinolones, aminoglycosides	[23]

IncI2	*mcr-1*	*E. coli*	Clinical	Korea	None	None	*$bla_{CTX\text{-}M\text{-}55}$, $bla_{NDM\text{-}1}$, bla_{TEM}, qepA1, rmtB*	[24]
IncX4	*mcr-1*	*E. coli, Klebsiella pneumoniae*	Clinical	Thailand	None	None	None	[25]
IncI2	*mcr-1*	*E. coli and K. pneumoniae*	Clinical	Thailand	None	None	None	[25]
IncHI2/IncN	*mcr-1*	*E. coli and K. pneumoniae*	Clinical	Thailand	IS*Apl1*	Tn6330	*$bla_{TEM\text{-}1B}$, $bla_{TEM\text{-}135}$*	[25]
IncX4	*mcr-1*	*S. enterica*	Retail meat	Brazil	IS26	None	Aminoglycosides, β-lactams, fluoroquinolones, sulfonamides, tetracyclines	[26]
Hybrid (IncHI2, IncX1)	*mcr-1.1, mcr-3.1*	*E. coli*	Swine feces	China	Not specified	Not specified	Not specified	[27]
IncX4	*mcr-1.1*	*E. coli*	Clinical	Brazil	None	None	*$bla_{TEM\text{-}1A}$, aph(6)-Id, aph(3'')-Ib, qnrB19*	[28]
IncX4	*mcr-1.1*	*E. coli*	Clinical	Brazil	None	None	*$bla_{CTX\text{-}M\text{-}2}$, aadA1, aac(3)-IId, gyrA mutations*	[28]
Hybrid (IncHI2, IncFIB, etc)	*mcr-1*	*E. coli*	Clinical	China	IS*Apl1*	Not specified	*bla_{TEM}, ant(3')-I, aph(4)-Ia, aph(3')-I, aac(6')-Ib, oqxA, fosA3, $bla_{CTX\text{-}M}$*	[29]
Hybrid (IncP, IncFII)	*mcr-1*	*E. coli*	Clinical	China	Not specified	Not specified	*bla_{TEM}, ant(3')-I, aph(4)-Ia, oqxA, oqxB*	[29]

IncI2	*mcr-1*	*E. coli*	Chicken farm	China	IS1	Not specified	β-lactams, sulfonamides, tetracyclines, aminoglycosides	[3]
IncI2	*mcr-1*	*Enterobacteriaceae*	Human, animals, and environmental	Korea	IS*Apl1*, IS*Kpn26*, IS1294	None	Variable	[30]
IncX4	*mcr-1*	*Enterobacteriaceae*	Human, animals, and environmental	Korea	None	None	None	[30]
IncHI2	*mcr-1*	*E. coli*	Human, animals, and environmental	Korea	Not specified	Not specified	Beta-lactams, sulfonamides, quinolones	[30]
Hybrid (IncFIA, IncHI1A, etc)	*mcr-1, mcr-3.19*	*E. coli*	Not specified	China	IS26, ISVsa5	Tn6330, TnAs2	*strAB, erm(B), mph(A), bleO, aac(3)-IId, mer, aph(3)-Ia, mef(B), sul3, aadA1, cmlA, aadA2, dfrA12*	[31]
IncX4	*mcr-1*	*E. coli*	Pigs and humans	Thailand	IS26	Tn6330	*qnrS*	[31]
IncHI1	*mcr-1*	*E. coli*	Pigs	Thailand	IS1, ISKpn40	None	bla_{CTX-M}	[31]
IncX4	*mcr-1.1*	*E. coli*	Retail chicken	Netherlands	None	None	None	[32]
IncI2	*mcr-1*	*E. coli*	Public trash cans	China	None	None	$bla_{\text{CTX-M-55}}$	[33]
IncX4	*mcr-1.1*	*Salmonella enterica Serovar Cannstatt*	Clinical (fatal Sepsis Case)	Thailand	None	None	$bla_{\text{CTX-M-14}}$, *aac(3)IId, aac(69)Iaa, floR, qnrS1, sul2, tetA, tetM*	[34]
Hybrid (IncFIB, IncX1, etc)	*mcr-1*	*Acinetobacter baumannii*	Clinical	South Africa	IS*Apl1*, IS903B	Tn3, Tn7	*tet(M), cmlA1, qnrS1*	[35]

IncX4	*mcr-1*	*E. coli*	Fresh meat	Czech Republic	IS*Kpn26*	Not specified	*tet(M)*	[36]
IncI2	*mcr-1*	*E. coli*	Fresh meat	Czech Republic	IS*Kpn26*	Not specified	Variable	[36]
IncX4	*mcr-1.26*	*E. coli*	Poultry	Germany	IS26	Tn2	bla_{TEM}	[37]
IncX4	*mcr-1*	*E. coli*	Wild deer	Portugal	None	None	None	[38]
IncI2	*mcr-1*	*E. coli*	Clinical	Vietnam	IS*Apl1*	None	Variable	[39]
IncP	*mcr-1*	*E. coli*	Community and Hospital Settings	Vietnam	IS*Apl1*	None	Aminoglycosides, sulfonamides, beta-lactams	[39]
IncHI2	*mcr-1*	*E. coli*	Camel	Tunisia	Not specified	Not specified	$bla_{\text{CTX-M-1}}$	[4]
IncI1	*mcr-1*	*E. coli*	Camel	Tunisia	Not specified	None	$bla_{\text{CTX-M-15}}$	[4]
IncY	*mcr-1*	*E. coli*	Camel	Tunisia	Not specified	None	$bla_{\text{CTX-M-15}}$, tetracycline, nalidixic acid, sulfonamide-trimethoprim	[4]
IncY	*mcr-1*	*K. pneumoniae*	Camel	Tunisia	Not specified	None	$bla_{\text{CTX-M-15}}$, tetracycline, nalidixic acid, sulfonamide-trimethoprim	[4]
Hybrid (IncX1, IncFIA)	*mcr-1*	*E. coli*	Clinical	China	IS26, ISCR2	Not specified	*tet(X4)*	[40]
IncHI2	*mcr-1*	*E. coli*	Clinical	China	IS26	None	Multiple resistance genes (not detailed)	[40]
Hybrid (IncFII, IncI2)	*mcr-1*	*E. coli*	Not specified	China	Not specified	Not specified	$bla_{\text{CTX-M}}$	[41]

IncN1-IncHI2	*mcr-1*	*K. pneumoniae*	Chicken meat	China	None	None	*floR, tet(A),* bla_{TEM-1}, bla_{OXA-1}	[42]
IncX4	*mcr-1*	*S. enterica*	Pigs	China	None	None	None	[42]
IncHI2	*mcr-1*	*Salmonella Typhimurium*	Diarrheal outpatients	China	IS*Apl1*	Tn6330	$bla_{CTX-M-14}$, *oqxAB, fosA3, floR*	[43]
IncX4	*mcr-1*	*E. coli and K. pneumoniae*	Swine and chicken	China	None	None	None	[44]
IncI2	*mcr-1*	*E. coli and K. pneumoniae*	Human, Swine and chicken	China	None	None	None	[44]
IncX4	*mcr-1*	*E. coli*	Pigeon feces	China	None	None	None	[45]
IncI2	*mcr-1*	*Nontyphoidal Salmonella*	Poultry and humans	China	IS*Apl1*	None	blaTEM, tetA	[46]
IncX4	*mcr-1*	*Nontyphoidal Salmonella*	Poultry and humans	China	Not specified	None	Tetracyclines, aminoglycosides	[46]
IncI2	*mcr-1*	*E. coli*	Pig, pork, and human	China	IS*Apl1*	Tn6330	*tet(A), floR, sul2, aadA1, strA, strB*	[47]
IncX4	*mcr-1*	*E. coli*	Poultry and livestock	West Africa	IS26	Not specified	bla_{TEM-1}, *tetA*	[48]
IncHI2	*mcr-1*	*E. coli*	Chickens and pigs	China	IS*Apl1*	Tn6330	*floR*, $bla_{CTX-M-55}$, *fosA3*	[49]
IncX4	*mcr-1*	*E. coli*	Chickens and pigs	China	IS26	None	None	[49]
IncI2	*mcr-1*	*E. coli*	Retail chicken	China	IS*Apl1*	None	$bla_{CTX-M-55}$	[49]
IncI2	*mcr-1*	*E. coli*	Clinical	Uruguay	None	None	bla_{CMY-2}	[5]
IncHI2	*mcr-1*	*Salmonella Typhimurium*	Human fecal samples (hospital)	China	None	None	Sulfonamide, tetracycline resistance genes	[50]
IncI2	*mcr-1*	*Salmonella Typhimurium*	Human fecal samples (hospital)	China	None	None	*fosA3*	[50]

IncI2	*mcr-1.1*	*E. coli*	Clinical (prosthesis infection)	China	None	None	None	[51]
IncI2	*mcr-1*	*E. coli*	Clinical	Pakistan	None	None	$bla_{CTX-M-15}$, bla_{TEM-1}	[52]
IncX4	*mcr-1*	*K. pneumoniae*	Clinical (bloodstram infections)	China	None	None	β-lactams, aminoglycosides, quinolones, sulfonamides	[53]
IncX4	*mcr-1*	*Pseudomonas aeruginosa*	Pediatric patients	China	None	None	bla_{NDM-1}, bla_{KPC-1}, bla_{GES}	[54]
Hybrid (IncF53:A-:B-, phage-like)	*mcr-1.1*	*E. coli*	Clinical	China	IS26	Not specified	Not specified	[55]
IncI2	*mcr-1*	*E. coli*	Urban sewage	China	None	None	$bla_{CTX-M-199}$, aminoglycosides, tetracyclines	[56]
Hybrid (IncFIB, IncFIC)	*mcr-1*	*E. coli*	Clinical	China	Not specified	Not specified	bla_{CTX-M}, *aph(3')-Id, sul2*	[57]
IncX4	*mcr-1*	*E. coli*	Clinical	China	IS26	None	None	[57]
IncI2	*mcr-1*	*E. coli*	Clinical	China	None	None	None	[57]
Hybrid (IncA/C, IncFIB)	*mcr-1*	*K. pneumoniae*	Clinical	India	Not specified	Not specified	bla_{NDM}	[58]
IncX4	*mcr-1*	*Escherichia fergusonii*	Farm environment	China	IS26	None	*qnrS1*	[59]
IncI2	*mcr-1.1*	*Escherichia fergusonii*	Pig feces	China	None	None	None	[59]
IncI2	*mcr-1*	*E. coli*	Clinical	Philippines	None	None	$bla_{CTX-M-55}$, bla_{TEM-1B}, *sul2*	[6]
IncI2	*mcr-1*	*E. coli*	Clinical	China	IS*Apl1*	Tn6330	None	[60]
IncX4	*mcr-1*	*E. coli*	Clinical	China	IS26	None	None	[60]

Hybrid (IncR/IncN)	*mcr-8.2*	*K. pneumoniae*	Clinical	China	IS903B, ISEcl1	Not specified	*tet(A)*	[61]
IncX1	*mcr-1.1*	*K. pneumoniae*	Clinical	China	IS*Kpn26*	None	*tet(M)*	[61]
IncI2	*mcr-1*	*S. enterica*	Clinical	China	IS*Apl1*	Tn6330	None	[62]
IncX4	*mcr-1*	*S. enterica*	Clinical	China	None	Tn6330	None	[62]
IncC	*mcr-3*	*S. enterica*	Clinical	China	ISKpn40	Not speciied	$bla_{CTX-M-55}$, *aph(6)-Id, tet(A), catA2*	[62]
IncI2	*mcr-1.1*	*E. coli*	Meat	Thailand	IS*Apl1*	Tn6330	None	[63]
IncP1	*mcr-3.5*	*E. coli*	Meat	Thailand	IS4321, ISKpn40	None	*dgkA* (associated with lipid metabolism)	[63]
IncX4	*mcr-1*	*E. coli*	Meat	Thailand	No	None	None	[63]
IncX4	*mcr-1*	*E. coli*	Poultry	Romania	IS*Apl1*	Tn6330	bla_{TEM-1}, bla_{CMY-2}, *aac(3)-IIa, aph-3-Ia*	[64]
IncHI2	*mcr-1*	*Salmonella Typhimurium*	Clinical	China	IS*Apl1*	None	$bla_{CTX-M-14}$, *aac(3)-IVa, aadA2, aph(4)-Ia, sul1, sul2, sul3, floR*	[65]
IncX4	*mcr-1.1*	*E. coli*	Clinical	Poland	None	None	None	[66]
IncX4	*mcr-1.1*	*E. coli*	Clinical	Brazil	IS1†	None	*aph(3")-Ib, aph(6)-Id, sul2,* bla_{TEM1}	[67]
IncHI2A	*mcr-1.1*	*E. coli*	Clinical	Brazil	No	None	*aadA, aph(3")-ib, aph(6)-id, sul1,* bla_{TEM1}	[67]
IncX4	*mcr-1.1*	*E. coli*	Clinical	Brazil	None	None	*aac(3)-IIb, aph(3")-Ib, aph(6)-Id, floR, sul2,* bla_{TEM1}	[67]
IncX4	*mcr-1*	*E. coli*	Clinical	China	IS26	None	None	[68]

IncI2	*mcr-1*	*E. coli*	Clinical	China	None	None	Variable	[68]
IncHI2	*mcr-1*	*E. coli*	Swine origin	China	IS*Apl1*	None	$bla_{TEM\text{-}1A}$, *aac(3)-IIa, sul1, sul2, dfrA1, tet(A)*	[69]
IncX4	*mcr-1*	*E. coli*	Swine origin	China	None	None	None	[69]
IncI2	*mcr-1*	*E. coli*	Swine origin	China	None	None	Variable	[69]
IncHI1	*mcr-1*	*K. pneumoniae*	Clinical	Taiwan	IS*Apl1*	Tn6390	*aadA1, aadA2, aph(3")-Ia, aac(3)-IIa, aph(3")-Ib, aph(6)-Id,* $bla_{TEM\text{-}1B}$, *cmlA1, sul3*	[7]
IncHI2	*mcr-1*	*K. pneumoniae*	Clinical	Taiwan	IS*Apl1*	Tn6330	*aadA1, aadA2, aph(3')-Ia,* $bla_{CTX\text{-}M\text{-}14}$, *oqxAB, fosA3, cmlA1, sul1, sul3, dfrA12*	[7]
IncHI1	*mcr-1*	*K. pneumoniae*	Clinical	Taiwan	IS*Apl1*	Tn6330	*aadA1, aadA8b,* $bla_{CMY\text{-}2}$, *lnu(F), floR, sul3, tet(M)*	[7]
IncX4	*mcr-1*	*K. pneumoniae*	Clinical	Taiwan	None	None	None	[7]
IncHI2	*mcr-1*	*Salmonella enterica Serovar Sinstorf*	Clinical (diarrheal patients	China	Not specified	Not specified	$bla_{CTX\text{-}M\text{-}14}$, *qnrS2*, ESBL genes	[70]
IncI2	*mcr-1*	*Salmonella enterica Serovar Sinstorf*	Clinical (diarrheal patients	China	None	None	$bla_{TEM\text{-}1}$	[70]
IncX4	*mcr-1*	*E. coli*	Clinical	Netherlands	IS26	None	Unspecified	[71]
IncI2	*mcr-1*	*E. coli and K. pneumoniae*	Clinical	Netherlands	Not specified	None	ESBLs	[71]

Hybrid (IncI1, IncHI, etc)	*mcr-1*	*E. coli*	Clinical	Taiwan	IS*Apl1*	Tn7511	*aph(3'')-Ib, aph(6)-Id, aph(3')-Ia, aac(3)-IIe, aadA1, aadA2, bla*$_{TEM-1}$*, cmlA1, qacL, sul3*	[72]
IncX4	*mcr-1*	*Salmonella enterica Serotype Typhimurium*	Children and pork offal	China	IS26	None	None	[73]
IncI2	*mcr-1*	*Salmonella enterica Serotype Typhimurium*	Children and pork offal	China	IS*Apl1*	None	None	[73]
IncHI2	*mcr-1*	*E. coli*	Goats	France	IS*Apl1*	None	*bla*$_{TEM-1A}$*, aac(3)-IIa, sul1, sul2, dfrA1, tet(A)*	[74]
IncX4	*mcr-1*	*E. coli*	Goats	France	None	None	None	[74]
IncX4	*mcr-2*	*E. coli*	Swine	Belgium/ Germany	ISEc69	None	None	[75]
IncP1-like	*mcr-2*	*E. coli*	Swine	Belgium	ISEc69	None	None specified	[75]
IncI2	*mcr-1*	*E. coli*	Poultry farm	China	IS683, IS2	None	*bla*$_{CTX-M-64}$*, bla*$_{TEM-1}$*, qnrS*	[76]
IncHI2	*mcr-1*	*E. coli*	Poultry farm	China	IS*Apl1*, IS2	ISEcp1	*bla*$_{CTX-M-14}$*, qnrS2, bla*$_{CTX-M-64}$	[76]
Hybrid (IncFII, IncFIA, etc)	*mcr-1*	*E. coli*	Clinical	China	IS26, IS15DI, IS5075	Not specified	*aac(3)-IV, aph(3')-Ia, aph(4)-Ia, mph(A), bla*$_{CTX-M-14}$	[77]
IncX4	*mcr-1*	*E. coli*	Clinical	Czech Republic	None	None	None	[78]

IncI2	*mcr-1.1*	*E. coli*	Clinical	Czech Republic	None	None	None	[78]
IncI2	*mcr-1*	*E. coli*	Clinical	China	None	None	$bla_{CTX-M-55, -64, -132, -199}$	[79]
IncI2	*mcr-1*	*E. coli*	Poultry	Nigeria	Not specified	None	ESBLs	[8]
Hybrid (IncFIA(HI1), IncHI2, etc)	*mcr-1*	*K. pneumoniae*	Clinical	China	IS*Apl1*	Tn6330-like	β-lactams, sulfonamides, aminoglycosides	[80]
Hybrid (IncFIB/IncHI1B)	*mcr-1*	*K. pneumoniae*	Clinical	China	IS*Apl1*, ISEc33	Tn6330-like	β-lactams, aminoglycosides	[80]
IncI2	*mcr-1.1*	*E. coli*	Clinical	China	None	None	$bla_{CTX-M-65}$, bla_{OXA-1}, *dfrA14, qnrS1, cmlA5, sul1*	[81]
IncX4	*mcr-1.1*	*E. coli*	Pigs	China	None	None	None	[82]
Phage-like p0111	*mcr-1.1*	*E. coli*	Pigs	China	None	None	None	[82]
IncHI2	*mcr-1*	*E. coli*	Clinical	Czech Republic	IS*Apl1*	None	*aadA1, aph(3')-Ia, cmlA1, tetA, sul3*	[83]
IncX4	*mcr-1*	*E. coli*	Clinical	Czech Republic	None	None	Ampicillin, tetracycline	[83]
IncHI2	*mcr-1*	*E. coli*	Bovine mastitis	France	IS*Apl1*	Tn6330	*sul1, sul2, sul3, tet(A), tet(M)*	[84]
IncX4	-	*E. coli*	Bovine diarrhea	France	None	None	None	[84]
IncX4	*mcr-1.1/1.2*	*Salmonella Infantis*	Broiler meat	Italy	None	None	$bla_{CTX-M-1}$, *aac(6')-Iaa, gyrA, parC* mutations	[85]
IncX4	*mcr-1*	*E. coli*	Poultry	Germany	Not specified	Not specified	Unspecified	[86]

IncI2	*mcr-1*	*E. coli*	Poultry	Qatar	Not specified	None	β-lactams, fluoroquinolones, tetracyclines	[86]
Hybrid (IncHI1A:IncHI1B)	*mcr-1*	*E. coli*	Chicken farm	Peru	IS*Apl1*	Tn6330	*bla*$_{CTX-M-55}$	[87]
IncI2	*mcr-1*	*K. pneumoniae*	Chicken farm	Peru	None	None	None	[87]
IncX4	*mcr-1*	*E. coli*	Duck	Hungary	None	None	None	[88]
IncHI2/HI2A	*mcr-1*	*Salmonella Typhimurium*	Fresh eggs	China	IS*Apl1*	None	*aac(3)-IV, aph(4)-Ia, sul1, sul2, aac(6')-Ib-cr, bla*$_{OXA-1}$*, bla*$_{TEM-1B}$	[89]
IncX4	*mcr-1*	*E. coli*	Clinical	China	None	None	*aadA1,* aph(3′)-Ia, bla$_{CTX-M}$, dfrA5, aph(6)-Id, *aac(6')-Ib*	[9]
IncI2	*mcr-1*	*E. coli*	Clinical	China	None	None	aac(6')-Ib, *aph(6')-Ia, bla*$_{TEM-1}$*, mphA, sul2, oqxA, oqxB*	[9]
IncHI2	*mcr-1*	*E. coli, Salmonella*	Clinical	China	IS*Apl1*	None	*bla*$_{CTX-M-14}$*, aac(3)-IV, sul1, sul2, sul3, dfrA12*	[9]
IncX4	*mcr-1*	*E. coli*	Swine, poultry, and beef	Dominican Republic	IS26, IS*Apl1*	None	Aminoglycosides, quinolones, β-lactams, macrolides, tetracyclines	[90]
IncX4	*mcr-1.1/1.2*	*E. coli*	Raw meat	Italy	None	None	gyrA, tet(A), *bla*$_{TEM-1b}$	[91]

IncX4	*mcr-1*	*E. coli*	Pigs	Croatia	IS26	None	bla_{TEM-1B}, $bla_{CTX-M-1}$, *aac(3)-IId, aph(3')-Ia, aadA5, sul2, catA1, gyrA, parC*	[92]
IncI2	*mcr-1*	*E. coli*	Broiler farm	China	IS*Apl1*	None	β-lactams, aminoglycosides	[93]
IncX4	*mcr-1*	*E. coli*	Broiler farm	China	IS*Kpn26*	None	*tetA*	[93]
IncX4	*mcr-1.1*	*E. coli*	Clinical sample	Romania	None	None	β-lactams, aminoglycosides	[94]
IncI2	*mcr-1*	*E. coli*	Retail raw chicken	Korea	None	None	None	[95]
IncI2	*mcr-1*	*E. coli*	Retail raw chicken	Korea	None	None	None	[95]
IncHI2	*mcr-1*	*E. coli*	Lettuce	Portugal	IS26	Tn2	bla_{TEM-1}, *aadA1, aph(4)-Ia, aph(6)-Id, aac(3)-Iv, mdf(A), floR-type, tetA, sul2*	[96]
IncX4	*mcr-1.1*	*E. coli*	Rainbow trout aquaculture	Lebanon	None	None	*aac(3)-IId, aadA2, ant(3'')-Ia, aph(3')-Ia,* bla_{TEM-1B}, *dfrA12, erm42, floR, mdf(A), mph(A), sul1, sul2, tetA, strA*	[97]
IncHI2	*mcr-1*	*E. coli*	Chicken carcasses	Egypt	IS*Apl1*	Tn6330	Tetracycline, sulfonamides, chloramphenicol	[98]
IncX4	*mcr-1*	*E. coli*	Chicken carcasses	Egypt	None	None	None	[98]

IncI2	*mcr-1*	*E. coli*	Chicken carcasses	Egypt	None	None	Amoxicillin, ticarcillin, sulfonamides, tetracycline, chloramphenicol	[98]
IncI2	*mcr-1*	*Salmonella Typhimurium*	Healthy pig	Korea	None	None	Ampicillin, chloramphenicol, gentamicin, streptomycin, sulfonamides, tetracycline	[99]
IncX4	*mcr-1*	*E. coli*	River water	Taiwan	IS26	Tn3	None	[100]
IncHI2	*mcr-1.1*	*E. coli*	Clinical	Egypt	IS*Apl1*	Tn21	bla_{SHV}, bla_{TEM}, *aac(3)-IIa, aph(6)-Id, tetA, sul2, sul3, qnrS1, floR, dfrA1*	[101]
IncX4	*mcr-1.1*	*E. coli*	Pig farm	China	None	None	None	[102]

References

1. Tang, B.; Chang, J.; Zhang, L.; Liu, L.; Xia, X.; Hassan, B.H.; Jia, X.; Yang, H.; Feng, Y. Carriage of Distinct Mcr-1-Harboring Plasmids by Unusual Serotypes of Salmonella. *Adv. Biosyst.* **2020**, *4*, 1900219, doi:10.1002/adbi.201900219.

2. Ali, A.; Fontana, H.; Sano, E.; Li, R.; Humayon, M.; Lincopan, N.; Mohsin, M. Genomic Features of a High-Risk Mcr-1.1-Positive Escherichia Coli ST10 Isolated from Cattle Farm Environment. *Environ. Sci. Pollut. Res.* **2021**, *28*, doi:10.1007/s11356-021-15437-6.
3. Li, X.-P.; Sun, R.-Y.; Song, J.-Q.; Fang, L.-X.; Zhang, R.-M.; Lian, X.-L.; Liao, X.-P.; Liu, Y.-H.; Lin, J.; Sun, J. Within-Host Heterogeneity and Flexibility of *Mcr-1* Transmission in Chicken Gut. *Int. J. Antimicrob. Agents* **2020**, *55*, 105806, doi:10.1016/j.ijantimicag.2019.09.010.
4. Saidani, M.; Messadi, L.; Mefteh, J.; Chaouechi, A.; Soudani, A.; Selmi, R.; Dâaloul-Jedidi, M.; Ben Chehida, F.; Mamlouk, A.; Jemli, M.H.; et al. Various Inc-Type Plasmids and Lineages of *Escherichia Coli* and *Klebsiella Pneumoniae* Spreading *Bla*CTX-M-15, *Bla*CTX-M-1 and *Mcr-1* Genes in Camels in Tunisia. *J. Glob. Antimicrob. Resist.* **2019**, *19*, 280–283, doi:10.1016/j.jgar.2019.05.007.
5. Papa-Ezdra, R.; Grill Diaz, F.; Vieytes, M.; García-Fulgueiras, V.; Caiata, L.; Ávila, P.; Brasesco, M.; Christophersen, I.; Cordeiro, N.F.; Algorta, G.; et al. First Three Escherichia Coli Isolates Harbouring Mcr-1 in Uruguay. *J. Glob. Antimicrob. Resist.* **2020**, *20*, 187–190, doi:10.1016/j.jgar.2019.07.016.
6. Velasco, J.M.S.; Valderama, M.T.G.; Margulieux, K.R.; Diones, P.C.S.; Reyes, A.M.B.; Leonardia, S.G.; Liao, C.P.; Chua, D.A.; Navarro, F.C.S.; Ruekit, S.; et al. First Report of the Mcr-1 Colistin Resistance Gene Identified in Two Escherichia Coli Isolates from Clinical Samples, Philippines, 2018. *J. Glob. Antimicrob. Resist.* **2020**, *21*, 291–293, doi:10.1016/j.jgar.2019.12.018.
7. Lin, Y.-C.; Kuroda, M.; Suzuki, S.; Mu, J.-J. Emergence of the Mcr-1 Colistin Resistance Gene in Extended-Spectrum β-Lactamase-Producing Klebsiella Pneumoniae in Taiwan. *J. Glob. Antimicrob. Resist.* **2021**, *24*, 278–284, doi:10.1016/j.jgar.2020.12.024.
8. Anyanwu, M.U.; Marrollo, R.; Paolucci, M.; Brovarone, F.; Nardini, P.; Chah, K.F.; Shoyinka, S.V.O.; Carretto, E. Isolation and Characterisation of Colistin-Resistant Enterobacterales from Chickens in Southeast Nigeria. *J. Glob. Antimicrob. Resist.* **2021**, *26*, 93–100, doi:10.1016/j.jgar.2021.04.030.
9. Xie, J.; Liang, B.; Xu, X.; Yang, L.; Li, H.; Li, P.; Qiu, S.; Song, H. Identification of Mcr-1-Positive Multidrug-Resistant Escherichia Coli Isolates from Clinical Samples in Shanghai, China. *J. Glob. Antimicrob. Resist.* **2022**, *29*, 88–96, doi:10.1016/j.jgar.2022.02.008.
10. Protonotariou, E.; Meletis, G.; Malousi, A.; Kotzamanidis, C.; Tychala, A.; Mantzana, P.; Theodoridou, K.; Ioannidou, M.; Hatzipantelis, E.; Tsakris, A.; et al. First Detection of Mcr-1-Producing Escherichia Coli in Greece. *J. Glob. Antimicrob. Resist.* **2022**, *31*, 252–255, doi:10.1016/j.jgar.2022.10.008.
11. Lee, S.; An, J.-U.; Kim, W.-H.; Yi, S.; Lee, J.; Cho, S. Different Threats Posed by Two Major Mobilized Colistin Resistance Genes - Mcr-1.1 and Mcr-3.1 - Revealed through Comparative Genomic Analysis. *J. Glob. Antimicrob. Resist.* **2023**, *32*, 50–57, doi:10.1016/j.jgar.2022.12.007.
12. Zając, M.; Iwan, E.; Skarżyńska, M.; Kwit, R.; Skóra, M.; Lalak, A.; Śmiałowska-Węglińska, A.; Kamińska, E.; Pietruk, M.; Wasyl, D. The First Description of the Complete Genome Sequence of Multidrug-Resistant Salmonella Enterica Serovar Monophasic Typhimurium (1,4,[5],12:I:-) Isolate with the Mcr-1.1 Gene on IncHI2 Found in Pig in Poland. *J. Glob. Antimicrob. Resist.* **2023**, *33*, 218–220, doi:10.1016/j.jgar.2023.04.008.
13. Feng, J.; Zhuang, Y.; Luo, J.; Xiao, Q.; Wu, Y.; Chen, Y.; Chen, M.; Zhang, X. Prevalence of Colistin-Resistant *Mcr-1*-Positive *Escherichia Coli* Isolated from Children Patients with Diarrhoea in Shanghai, 2016–2021. *J. Glob. Antimicrob. Resist.* **2023**, *34*, 166–175, doi:10.1016/j.jgar.2023.06.006.
14. Patil, S.; Pai, L.; Chen, X.; Francisco, N.M.; Chen, H.; Chen, Y.; Dong, S.; Liu, S.; Wen, F. Genomic Characterisation of Multi-Drug Resistant Escherichia Coli and Klebsiella Pneumoniae Co-Harbouring Mcr-1 and Mcr-3 Genes on a Single Plasmid from Paediatric Clinical Cases. *J. Glob. Antimicrob. Resist.* **2023**, *34*, 134–140, doi:10.1016/j.jgar.2023.07.012.
15. Zhang, X.; Peng, L.; Ke, Y.; Zhao, D.; Yu, G.; Zhou, Y.; Li, X.; Weng, X. Emergence of a Clinical Isolate of E. Coil ST297 Co-Carrying blaNDM-13 and Mcr-1.1 in China. *J. Infect. Public Health* **2023**, *16*, 1813–1820, doi:10.1016/j.jiph.2023.09.007.

16. Furlan, J.P.R.; Lopes, R.; Ramos, M.S.; Dos Santos, L.D.R.; da Silva Rosa, R.; Savazzi, E.A.; Stehling, E.G. Colistin-Resistant Mcr-1-Positive Escherichia Coli ST1775-H137 Co-Harboring blaCTX-M-2 and blaCMY-2 Recovered from an Urban Stream. *Infect. Genet. Evol. J. Mol. Epidemiol. Evol. Genet. Infect. Dis.* **2021**, *96*, 105156, doi:10.1016/j.meegid.2021.105156.
17. Yang, C.; Chen, K.; Ye, L.; Heng, H.; Chan, E.W.C.; Chen, S. Genetic and Drug Susceptibility Profiles of Mcr-1-Bearing Foodborne Salmonella Strains Collected in Shenzhen, China during the Period 2014-2017. *Microbiol. Res.* **2022**, *265*, 127211, doi:10.1016/j.micres.2022.127211.
18. Liu, K.-D.; Jin, W.-J.; Li, R.-B.; Zhang, R.-M.; Sun, J.; Liu, Y.-H.; Wang, M.-G.; Liao, X.-P. Prevalence and Molecular Characteristics of Mcr-1-Positive Escherichia Coli Isolated from Duck Farms and the Surrounding Environments in Coastal China. *Microbiol. Res.* **2023**, *270*, 127348, doi:10.1016/j.micres.2023.127348.
19. Wang, Z.; Jiang, Z.; Xu, H.; Jiao, X.; Li, Q. Prevalence and Molecular Characterization of Mcr-1-Positive Foodborne ST34-Salmonella Isolates in China. *Microbiol. Res.* **2023**, *274*, 127441, doi:10.1016/j.micres.2023.127441.
20. Li, W.; Li, Y.; Jia, Y.; Sun, H.; Zhang, C.; Hu, G.; Yuan, L. Genomic Characteristics of *Mcr-1* and *Bla*CTX-M-Type in a Single Multidrug-Resistant *Escherichia Coli* ST93 from Chicken in China. *Poult. Sci.* **2021**, *100*, 101074, doi:10.1016/j.psj.2021.101074.
21. Wang, Z.; Fu, Y.; Schwarz, S.; Yin, W.; Walsh, T.R.; Zhou, Y.; He, J.; Jiang, H.; Wang, Y.; Wang, S. Genetic Environment of Colistin Resistance Genes Mcr-1 and Mcr-3 in Escherichia Coli from One Pig Farm in China. *Vet. Microbiol.* **2019**, *230*, 56–61, doi:10.1016/j.vetmic.2019.01.011.
22. Jiang, L.; Zhu, H.; Wei, J.; Jiang, L.; Li, Y.; Li, R.; Wang, Z.; Wang, M. Enterobacteriaceae Genome-Wide Analysis Reveals Roles for P1-like Phage-Plasmids in Transmission of Mcr-1, tetX4 and Other Antibiotic Resistance Genes. *Genomics* **2023**, *115*, 110572, doi:10.1016/j.ygeno.2023.110572.
23. Feng, Y. Transferability of MCR-1/2 Polymyxin Resistance: Complex Dissemination and Genetic Mechanism. *ACS Infect. Dis.* **2018**, *4*, 291–300, doi:10.1021/acsinfecdis.7b00201.
24. Han, S.; Kim, J.S.; Hong, C.-K.; Park, S.-H.; Kim, H.S.; Yu, J.K.; Park, J.; Kim, J.; Lee, S.-M.; Oh, Y.-H. Identification of an Extensively Drug-Resistant Escherichia Coli Clinical Strain Harboring Mcr-1 and blaNDM-1 in Korea. *J. Antibiot. (Tokyo)* **2020**, *73*, 852–858, doi:10.1038/s41429-020-0350-1.
25. Boueroy, P.; Wongsurawat, T.; Jenjaroenpun, P.; Chopjitt, P.; Hatrongjit, R.; Jittapalapong, S.; Kerdsin, A. Plasmidome in Mcr-1 Harboring Carbapenem-Resistant Enterobacterales Isolates from Human in Thailand. *Sci. Rep.* **2022**, *12*, 19051, doi:10.1038/s41598-022-21836-7.
26. Rau, R.B.; de Lima-Morales, D.; Wink, P.L.; Ribeiro, A.R.; Barth, A.L. Salmonella Enterica Mcr-1 Positive from Food in Brazil: Detection and Characterization. *Foodborne Pathog. Dis.* **2020**, *17*, 202–208, doi:10.1089/fpd.2019.2700.
27. Sun, D.; Jin, S.; Wang, J.; Wang, Z.; Fan, J.; Xu, Z.; Xu, Y.; Chen, X.; Jiao, X. Multidrug-Resistant Escherichia Coli Strain Isolated from Swine in China Harbors Mcr-3.1 on a Plasmid of the IncX1 Type That Cotransfers with Mcr-1.1. *Foodborne Pathog. Dis.* **2020**, *17*, 597–601, doi:10.1089/fpd.2019.2769.
28. Zamparette, C.P.; Schorner, M.; Campos, E.; Moura, Q.; Cerdeira, L.; Tartari, D.C.; Sereia, A.F.R.; Cunha, P.; Fontana, H.; de Oliveira, L.F.V.; et al. IncX4 Plasmid-Mediated Mcr-1.1 in Polymyxin-Resistant Escherichia Coli from Outpatients in Santa Catarina, Southern Brazil. *Microb. Drug Resist. Larchmt. N* **2020**, *26*, 1326–1333, doi:10.1089/mdr.2019.0203.
29. Liang, Z.; Pang, J.; Hu, X.; Nie, T.; Lu, X.; Li, X.; Wang, X.; Li, C.; Yang, X.; You, X. Low Prevalence of Mcr-1 Among Clinical Enterobacteriaceae Isolates and Co-Transfer of Mcr-1 and blaNDM-1 from Separate Donors. *Microb. Drug Resist. Larchmt. N* **2021**, *27*, 476–484, doi:10.1089/mdr.2020.0212.

30. Kim, Y.-J.; Seo, K.-H.; Kim, S.; Bae, S. Phylogenetic Comparison and Characterization of an Mcr-1-Harboring Complete Plasmid Genome Isolated from Enterobacteriaceae. *Microb. Drug Resist. Larchmt. N* **2022**, *28*, 492–497, doi:10.1089/mdr.2021.0164.
31. Leangapichart, T.; Stosic, M.S.; Hickman, R.A.; Lunha, K.; Jiwakanon, J.; Angkititrakul, S.; Magnusson, U.; Van Boeckel, T.P.; Järhult, J.D.; Sunde, M. Exploring the Epidemiology of *Mcr* Genes, Genetic Context and Plasmids in *Enterobacteriaceae* Originating from Pigs and Humans on Farms in Thailand. *J. Antimicrob. Chemother.* **2023**, *78*, 1395–1405, doi:10.1093/jac/dkad097.
32. Jamin, C.; Sanders, B.K.; Zhou, M.; Costessi, A.; Duijsings, D.; Kluytmans, J.A.J.W.; van Alphen, L.B.; Schrauwen, E.J.A. Genetic Analysis of Plasmid-Encoded Mcr-1 Resistance in Enterobacteriaceae Derived from Poultry Meat in the Netherlands. *JAC-Antimicrob. Resist.* **2021**, *3*, dlab156, doi:10.1093/jacamr/dlab156.
33. Long, X.; Li, J.; Yang, H.; Gao, Y.; Ma, J.; Zeng, X.; Tang, B. The Bla NDM-1 and Mcr-1 Genes Coexist in Escherichia Coli Strain Isolated from Public Trash Cans. *JAC-Antimicrob. Resist.* **2024**, *6*, dlae132, doi:10.1093/jacamr/dlae132.
34. Paveenkittiporn, W.; Kamjumphol, W.; Kerdsin, A. Draft Genome Sequence of Invasive Salmonella Enterica Serovar Cannstatt Harboring Mcr-1.1, Isolated from a Fatal Sepsis Case. *Microbiol. Resour. Announc.* **2021**, *10*, e01270-20, doi:10.1128/MRA.01270-20.
35. Mmatli, M.; Mbelle, N.M.; Osei Sekyere, J. Plasmid-Borne Mcr-1 and Replicative Transposition of Episomal and Chromosomal blaNDM-1, blaOXA-69, and blaOXA-23 Carbapenemases in a Clinical Acinetobacter Baumannii Isolate. *mSystems* **2025**, *10*, e0168324, doi:10.1128/msystems.01683-24.
36. Sismova, P.; Sukkar, I.; Kolidentsev, N.; Palkovicova, J.; Chytilova, I.; Bardon, J.; Dolejska, M.; Nesporova, K. Plasmid-Mediated Colistin Resistance from Fresh Meat and Slaughtered Animals in the Czech Republic: Nation-Wide Surveillance 2020–2021. *Microbiol. Spectr.* **2023**, *11*, e00609-23, doi:10.1128/spectrum.00609-23.
37. Binsker, U.; Oelgeschläger, K.; Neumann, B.; Werner, G.; Käsbohrer, A.; Hammerl, J.A. Genomic Evidence of Mcr-1.26 IncX4 Plasmid Transmission between Poultry and Humans. *Microbiol. Spectr.* **2023**, *11*, e0101523, doi:10.1128/spectrum.01015-23.
38. Dantas Palmeira, J.; V Cunha, M.; Ferreira, H.; Fonseca, C.; Tinoco Torres, R. Worldwide Disseminated IncX4 Plasmid Carrying Mcr-1 Arrives to Wild Mammal in Portugal. *Microbiol. Spectr.* **2022**, *10*, e0124522, doi:10.1128/spectrum.01245-22.
39. Vu Thi Ngoc, B.; Le Viet, T.; Nguyen Thi Tuyet, M.; Nguyen Thi Hong, T.; Nguyen Thi Ngoc, D.; Le Van, D.; Chu Thi, L.; Tran Huy, H.; Penders, J.; Wertheim, H.; et al. Characterization of Genetic Elements Carrying Mcr-1 Gene in Escherichia Coli from the Community and Hospital Settings in Vietnam. *Microbiol. Spectr.* **2022**, *10*, e0135621, doi:10.1128/spectrum.01356-21.
40. Lu, X.; Xiao, X.; Liu, Y.; Li, R.; Wang, Z. Emerging Opportunity and Destiny of Mcr-1- and Tet(X4)-Coharboring Plasmids in Escherichia Coli. *Microbiol. Spectr.* **2021**, *9*, e01520-21, doi:10.1128/Spectrum.01520-21.
41. He, K.; Li, W.; Zhao, B.; Xu, H.; Pan, Y.; He, D.; Hu, G.; Wu, H.; Yuan, L. Spreading Advantages of Coresident Plasmids blaCTX-M-Bearing IncFII and Mcr-1-Bearing IncI2 in Escherichia Coli. *Microbiol. Spectr.* **2022**, *10*, e01706-21, doi:10.1128/spectrum.01706-21.
42. Mei, C.-Y.; Jiang, Y.; Ma, Q.-C.; Lu, M.-J.; Wu, H.; Wang, Z.-Y.; Jiao, X.; Wang, J. Chromosomally and Plasmid-Located Mcr in Salmonella from Animals and Food Products in China. *Microbiol. Spectr.* **2022**, *10*, e02773-22, doi:10.1128/spectrum.02773-22.
43. Sun, R.-Y.; Fang, L.-X.; Ke, B.-X.; Sun, J.; Wu, Z.-W.; Feng, Y.-J.; Liu, Y.-H.; Ke, C.-W.; Liao, X.-P. Carriage and Transmission of Mcr-1 in Salmonella Typhimurium and Its Monophasic 1,4,[5],12:I:- Variants from Diarrheal Outpatients: A 10-Year Genomic Epidemiology in Guangdong, Southern China. *Microbiol. Spectr.* **2023**, *11*, e0311922, doi:10.1128/spectrum.03119-22.

44. Liu, Y.-Y.; Zhu, X.-Q.; Nang, S.; Xun, H.; Lv, L.; Yang, J.; Liu, J.-H. Greater Invasion and Persistence of Mcr-1-Bearing Plasmids in Escherichia Coli than in Klebsiella Pneumoniae. *Microbiol. Spectr.* **2023**, *11*, e0322322, doi:10.1128/spectrum.03223-22.
45. Lu, X.; Zhang, W.; Mohsin, M.; Wang, M.; Li, J.; Wang, Z.; Li, R. The Prevalence of Plasmid-Mediated Colistin Resistance Gene Mcr-1 and Different Transferability and Fitness of Mcr-1-Bearing IncX4 Plasmids in Escherichia Coli from Pigeons. *Microbiol. Spectr.* **2023**, *11*, e0363922, doi:10.1128/spectrum.03639-22.
46. Yang, T.; Li, W.; Cui, Q.; Qin, X.; Li, B.; Li, X.; Jia, H.; Yang, X.; Liu, C.; Wang, Y.; et al. Distribution and Transmission of Colistin Resistance Genes Mcr-1 and Mcr-3 among Nontyphoidal Salmonella Isolates in China from 2011 to 2020. *Microbiol. Spectr.* **2023**, *11*, e03833-22, doi:10.1128/spectrum.03833-22.
47. Lu, X.; Zhang, P.; Du, P.; Zhang, X.; Wang, J.; Yang, Y.; Sun, H.; Wang, Z.; Cui, S.; Li, R.; et al. Prevalence and Genomic Characteristics of Mcr-Positive Escherichia Coli Strains Isolated from Humans, Pigs, and Foods in China. *Microbiol. Spectr.* **2023**, *11*, e0456922, doi:10.1128/spectrum.04569-22.
48. Anyanwu, M.U.; Okpala, C.O.R.; Chah, K.F.; Shoyinka, V.S. Prevalence and Traits of Mobile Colistin Resistance Gene Harbouring Isolates from Different Ecosystems in Africa. *BioMed Res. Int.* **2021**, *2021*, 6630379, doi:10.1155/2021/6630379.
49. Mei, C.-Y.; Jiang, Y.; Ma, Q.-C.; Lu, M.-J.; Wu, H.; Wang, Z.-Y.; Jiao, X.; Wang, J. Low Prevalence of Mcr-1 in Escherichia Coli from Food-Producing Animals and Food Products in China. *BMC Vet. Res.* **2024**, *20*, 40, doi:10.1186/s12917-024-03891-6.
50. Lu, J.; Quan, J.; Zhao, D.; Wang, Y.; Yu, Y.; Zhu, J. Prevalence and Molecular Characteristics of Mcr-1 Gene in Salmonella Typhimurium in a Tertiary Hospital of Zhejiang Province. *Infect. Drug Resist.* **2019**, *12*, 105–110, doi:10.2147/IDR.S190269.
51. Li, Z.; Shi, R.; Wu, H.; Yan, P. First Identification of a Patient with Prosthesis-Related Infection Caused by an MCR-1.1-Producing ST131 Escherichia Coli After Rhinoplasty. *Infect. Drug Resist.* **2021**, *14*, 249–257, doi:10.2147/IDR.S295801.
52. Bilal, H.; Rehman, T.U.; Khan, M.A.; Hameed, F.; Jian, Z.G.; Han, J.; Yang, X. Molecular Epidemiology of Mcr-1, Bla KPC-2, and Bla NDM-1 Harboring Clinically Isolated Escherichia Coli from Pakistan. *Infect. Drug Resist.* **2021**, *14*, 1467–1479, doi:10.2147/IDR.S302687.
53. Ma, X.; Lv, X.; Feng, S.; Liu, R.; Fu, H.; Gao, F.; Xu, H. Genetic Characterization of an ST5571 Hypervirulent Klebsiella Pneumoniae Strain Co-Producing NDM-1, MCR-1, and OXA-10 Causing Bacteremia. *Infect. Drug Resist.* **2022**, *15*, 2293–2299, doi:10.2147/IDR.S360715.
54. Chen, H.; Mai, H.; Lopes, B.; Wen, F.; Patil, S. Novel Pseudomonas Aeruginosa Strains Co-Harbouring Bla NDM-1 Metallo β-Lactamase and Mcr-1 Isolated from Immunocompromised Paediatric Patients. *Infect. Drug Resist.* **2022**, *15*, 2929–2936, doi:10.2147/IDR.S368566.
55. Wu, R.; Lv, L.; Wang, C.; Gao, G.; Yu, K.; Cai, Z.; Liu, Y.; Yang, J.; Liu, J.-H. IS26-Mediated Formation of a Hybrid Plasmid Carrying Mcr-1.1. *Infect. Drug Resist.* **2022**, *15*, 7227–7234, doi:10.2147/IDR.S390765.
56. Zhang, Y.; Chen, J.; Yang, X.; Wu, Y.; Wang, Z.; Xu, Y.; Zhou, L.; Wang, J.; Jiao, X.; Sun, L. Emerging Mobile Colistin Resistance Gene Mcr-1 and Mcr-10 in Enterobacteriaceae Isolates From Urban Sewage in China. *Infect. Drug Resist.* **2025**, *18*, 1035–1048, doi:10.2147/IDR.S502067.
57. Liu, Y.; Wang, Q.; Qi, T.; Zhang, M.; Chen, R.; Si, Z.; Li, J.; Jin, Y.; Xu, Q.; Li, P.; et al. Molecular Epidemiology of mcr-1-Positive Polymyxin B-Resistant Escherichia coli Producing Extended-Spectrum β-Lactamase (ESBL) in a Tertiary Hospital in Shandong, China. *Pol. J. Microbiol.* **2024**, *73*, 363–375, doi:10.33073/pjm-2024-032.
58. Singh, S.; Pathak, A.; Rahman, M.; Singh, A.; Nag, S.; Sahu, C.; Prasad, K.N. Genetic Characterisation of Colistin Resistant Klebsiella Pneumoniae Clinical Isolates From North India. *Front. Cell. Infect. Microbiol.* **2021**, *11*, 666030, doi:10.3389/fcimb.2021.666030.

59. Liu, R.; Xu, H.; Guo, X.; Liu, S.; Qiao, J.; Ge, H.; Zheng, B.; Gou, J. Genomic Characterization of Two Escherichia Fergusonii Isolates Harboring Mcr-1 Gene From Farm Environment. *Front. Cell. Infect. Microbiol.* **2022**, *12*, 774494, doi:10.3389/fcimb.2022.774494.
60. Li, Q.; Qian, C.; Zhang, X.; Zhu, T.; Shi, W.; Gao, M.; Feng, C.; Xu, M.; Lin, H.; Lin, L.; et al. Colistin Resistance and Molecular Characterization of the Genomes of Mcr-1-Positive Escherichia Coli Clinical Isolates. *Front. Cell. Infect. Microbiol.* **2022**, *12*, 854534, doi:10.3389/fcimb.2022.854534.
61. Wang, Y.; Zhou, J.; Liu, H.; Wang, Q.; Zhang, P.; Zhu, J.; Zhao, D.; Wu, X.; Yu, Y.; Jiang, Y. Emergence of High-Level Colistin Resistance Mediated by Multiple Determinants, Including Mcr-1.1, Mcr-8.2 and crrB Mutations, Combined with Tigecycline Resistance in an ST656 Klebsiella Pneumoniae. *Front. Cell. Infect. Microbiol.* **2023**, *13*, 1122532, doi:10.3389/fcimb.2023.1122532.
62. Sun, X.; Zhang, L.; Meng, J.; Peng, K.; Huang, W.; Lei, G.; Wang, Z.; Li, R.; Yang, X. The Characteristics of Mcr-Bearing Plasmids in Clinical Salmonella Enterica in Sichuan, China, 2014 to 2017. *Front. Cell. Infect. Microbiol.* **2023**, *13*, 1240580, doi:10.3389/fcimb.2023.1240580.
63. Chopjitt, P.; Boueroy, P.; Morita, M.; Iida, T.; Akeda, Y.; Hamada, S.; Kerdsin, A. Genetic Characterization of Multidrug-Resistant Escherichia Coli Harboring Colistin-Resistant Gene Isolated from Food Animals in Food Supply Chain. *Front. Cell. Infect. Microbiol.* **2024**, *14*, 1289134, doi:10.3389/fcimb.2024.1289134.
64. Maciuca, I.E.; Cummins, M.L.; Cozma, A.P.; Rimbu, C.M.; Guguianu, E.; Panzaru, C.; Licker, M.; Szekely, E.; Flonta, M.; Djordjevic, S.P.; et al. Genetic Features of Mcr-1 Mediated Colistin Resistance in CMY-2-Producing Escherichia Coli From Romanian Poultry. *Front. Microbiol.* **2019**, *10*, 2267, doi:10.3389/fmicb.2019.02267.
65. Fan, J.; Zhang, L.; He, J.; Zhao, M.; Loh, B.; Leptihn, S.; Yu, Y.; Hua, X. Plasmid Dynamics of Mcr-1-Positive Salmonella Spp. in a General Hospital in China. *Front. Microbiol.* **2020**, *11*, 604710, doi:10.3389/fmicb.2020.604710.
66. Majewski, P.; Gutowska, A.; Smith, D.G.E.; Hauschild, T.; Majewska, P.; Hryszko, T.; Gizycka, D.; Kedra, B.; Kochanowicz, J.; Glowiński, J.; et al. Plasmid Mediated Mcr-1.1 Colistin-Resistance in Clinical Extraintestinal Escherichia Coli Strains Isolated in Poland. *Front. Microbiol.* **2021**, *12*, 547020, doi:10.3389/fmicb.2021.547020.
67. Girardello, R.; Piroupo, C.M.; Martins, J.; Maffucci, M.H.; Cury, A.P.; Franco, M.R.G.; Malta, F. de M.; Rocha, N.C.; Pinho, J.R.R.; Rossi, F.; et al. Genomic Characterization of Mcr-1.1-Producing Escherichia Coli Recovered From Human Infections in São Paulo, Brazil. *Front. Microbiol.* **2021**, *12*, 663414, doi:10.3389/fmicb.2021.663414.
68. Liu, Z.; Liu, Y.; Xi, W.; Liu, S.; Liu, J.; Mu, H.; Chen, B.; He, H.; Fan, Y.; Ma, W.; et al. Genetic Features of Plasmid- and Chromosome-Mediated Mcr-1 in Escherichia Coli Isolates From Animal Organs With Lesions. *Front. Microbiol.* **2021**, *12*, 707332, doi:10.3389/fmicb.2021.707332.
69. Cheng, P.; Yang, Y.; Cao, S.; Liu, H.; Li, X.; Sun, J.; Li, F.; Ishfaq, M.; Zhang, X. Prevalence and Characteristic of Swine-Origin Mcr-1-Positive Escherichia Coli in Northeastern China. *Front. Microbiol.* **2021**, *12*, 712707, doi:10.3389/fmicb.2021.712707.
70. Liu, G.; Qian, H.; Lv, J.; Tian, B.; Bao, C.; Yan, H.; Gu, B. Emergence of Mcr-1-Harboring Salmonella Enterica Serovar Sinstorf Type ST155 Isolated From Patients With Diarrhea in Jiangsu, China. *Front. Microbiol.* **2021**, *12*, 723697, doi:10.3389/fmicb.2021.723697.
71. Strepis, N.; Voor In 't Holt, A.F.; Vos, M.C.; Zandijk, W.H.A.; Heikema, A.P.; Hays, J.P.; Severin, J.A.; Klaassen, C.H.W. Genetic Analysis of Mcr-1-Carrying Plasmids From Gram-Negative Bacteria in a Dutch Tertiary Care Hospital: Evidence for Intrapatient and Interspecies Transmission Events. *Front. Microbiol.* **2021**, *12*, 727435, doi:10.3389/fmicb.2021.727435.

72. Cheng, Y.-H.; Chou, S.-H.; Huang, P.-H.; Yang, T.-C.; Juan, Y.-F.; Kreiswirth, B.N.; Lin, Y.-T.; Chen, L. Characterization of a Mcr-1 and CRISPR-Cas System Co-Harboring Plasmid in a Carbapenemase-Producing High-Risk ST11 Klebsiella Pneumoniae Strain. *Front. Microbiol.* **2021**, *12*, 762947, doi:10.3389/fmicb.2021.762947.
73. Zhang, H.; Xiang, Y.; Huang, Y.; Liang, B.; Xu, X.; Xie, J.; Du, X.; Yang, C.; Liu, H.; Liu, H.; et al. Genetic Characterization of Mcr-1-Positive Multidrug-Resistant Salmonella Enterica Serotype Typhimurium Isolated From Intestinal Infection in Children and Pork Offal in China. *Front. Microbiol.* **2021**, *12*, 774797, doi:10.3389/fmicb.2021.774797.
74. Treilles, M.; Châtre, P.; Drapeau, A.; Madec, J.-Y.; Haenni, M. Spread of the Mcr-1 Colistin-Resistance Gene in Escherichia Coli through Plasmid Transmission and Chromosomal Transposition in French Goats. *Front. Microbiol.* **2022**, *13*, 1023403, doi:10.3389/fmicb.2022.1023403.
75. Ewers, C.; Göpel, L.; Prenger-Berninghoff, E.; Semmler, T.; Kerner, K.; Bauerfeind, R. Occurrence of Mcr-1 and Mcr-2 Colistin Resistance Genes in Porcine Escherichia Coli Isolates (2010-2020) and Genomic Characterization of Mcr-2-Positive E. Coli. *Front. Microbiol.* **2022**, *13*, 1076315, doi:10.3389/fmicb.2022.1076315.
76. Tang, B.; Wang, J.; Zheng, X.; Chang, J.; Ma, J.; Wang, J.; Ji, X.; Yang, H.; Ding, B. Antimicrobial Resistance Surveillance of Escherichia Coli from Chickens in the Qinghai Plateau of China. *Front. Microbiol.* **2022**, *13*, 885132, doi:10.3389/fmicb.2022.885132.
77. Xia, S.; Wang, W.; Cheng, J.; Zhang, T.; Xia, Z.; Zhao, X.; Han, Y.; Li, Y.; Shi, X.; Qin, S. Emergence of a Novel Hybrid Mcr-1-Bearing Plasmid in an NDM-7-Producing ST167 Escherichia Coli Strain of Clinical Origin. *Front. Microbiol.* **2022**, *13*, 950087, doi:10.3389/fmicb.2022.950087.
78. Zelendova, M.; Papagiannitsis, C.C.; Sismova, P.; Medvecky, M.; Pomorska, K.; Palkovicova, J.; Nesporova, K.; Jakubu, V.; Jamborova, I.; Zemlickova, H.; et al. Plasmid-Mediated Colistin Resistance among Human Clinical Enterobacterales Isolates: National Surveillance in the Czech Republic. *Front. Microbiol.* **2023**, *14*, 1147846, doi:10.3389/fmicb.2023.1147846.
79. Sun, L.; Sun, G.-Z.; Jiang, Y.; Mei, C.-Y.; Wang, Z.-Y.; Wang, H.-Y.; Kong, G.-M.; Jiao, X.; Wang, J. Low Prevalence of Mobilized Resistance Genes blaNDM, Mcr-1, and Tet(X4) in Escherichia Coli from a Hospital in China. *Front. Microbiol.* **2023**, *14*, 1181940, doi:10.3389/fmicb.2023.1181940.
80. Zhao, Y.; Qian, C.; Ye, J.; Li, Q.; Zhao, R.; Qin, L.; Mao, Q. Convergence of Plasmid-Mediated Colistin and Tigecycline Resistance in Klebsiella Pneumoniae. *Front. Microbiol.* **2023**, *14*, 1221428, doi:10.3389/fmicb.2023.1221428.
81. Feng, J.; Wu, H.; Zhuang, Y.; Luo, J.; Chen, Y.; Wu, Y.; Fei, J.; Shen, Q.; Yuan, Z.; Chen, M. Stability and Genetic Insights of the Co-Existence of blaCTX-M-65, blaOXA-1, and Mcr-1.1 Harboring Conjugative IncI2 Plasmid Isolated from a Clinical Extensively-Drug Resistant Escherichia Coli ST744 in Shanghai. *Front. Public Health* **2023**, *11*, 1216704, doi:10.3389/fpubh.2023.1216704.
82. Guo, L.; Wang, J.; Wang, S.; Su, J.; Wang, X.; Zhu, Y. Genome Characterization of Mcr-1-Positive Escherichia Coli Isolated From Pigs With Postweaning Diarrhea in China. *Front. Vet. Sci.* **2020**, *7*, 503, doi:10.3389/fvets.2020.00503.
83. Tkadlec, J.; Kalova, A.; Brajerova, M.; Gelbicova, T.; Karpiskova, R.; Smelikova, E.; Nyc, O.; Drevinek, P.; Krutova, M. The Intestinal Carriage of Plasmid-Mediated Colistin-Resistant Enterobacteriaceae in Tertiary Care Settings. *Antibiot. Basel Switz.* **2021**, *10*, 258, doi:10.3390/antibiotics10030258.
84. Macori, G.; Nguyen, S.V.; Naithani, A.; Hurley, D.; Bai, L.; El Garch, F.; Woehrlé, F.; Miossec, C.; Roques, B.; O'Gaora, P.; et al. Characterisation of Early Positive Mcr-1 Resistance Gene and Plasmidome in Escherichia Coli Pathogenic Strains Associated with Variable Phylogroups under Colistin Selection. *Antibiot. Basel Switz.* **2021**, *10*, 1041, doi:10.3390/antibiotics10091041.

85. Casagrande Proietti, P.; Musa, L.; Stefanetti, V.; Orsini, M.; Toppi, V.; Branciari, R.; Blasi, F.; Magistrali, C.F.; Capomaccio, S.; Kika, T.S.; et al. Mcr-1-Mediated Colistin Resistance and Genomic Characterization of Antimicrobial Resistance in ESBL-Producing Salmonella Infantis Strains from a Broiler Meat Production Chain in Italy. *Antibiot. Basel Switz.* **2022**, *11*, 728, doi:10.3390/antibiotics11060728.
86. Al Mana, H.; Johar, A.A.; Kassem, I.I.; Eltai, N.O. Transmissibility and Persistence of the Plasmid-Borne Mobile Colistin Resistance Gene, Mcr-1, Harbored in Poultry-Associated E. Coli. *Antibiot. Basel Switz.* **2022**, *11*, 774, doi:10.3390/antibiotics11060774.
87. Carhuaricra, D.; Duran Gonzales, C.G.; Rodríguez Cueva, C.L.; Ignacion León, Y.; Silvestre Espejo, T.; Marcelo Monge, G.; Rosadio Alcántara, R.H.; Lincopan, N.; Espinoza, L.L.; Maturrano Hernández, L. Occurrence and Genomic Characterization of Mcr-1-Harboring Escherichia Coli Isolates from Chicken and Pig Farms in Lima, Peru. *Antibiot. Basel Switz.* **2022**, *11*, 1781, doi:10.3390/antibiotics11121781.
88. Szmolka, A.; Gellért, Á.; Szemerits, D.; Rapcsák, F.; Spisák, S.; Adorján, A. Emergence and Genomic Features of a Mcr-1 Escherichia Coli from Duck in Hungary. *Antibiot. Basel Switz.* **2023**, *12*, 1519, doi:10.3390/antibiotics12101519.
89. Li, C.; Gu, X.; Zhang, L.; Liu, Y.; Li, Y.; Zou, M.; Liu, B. The Occurrence and Genomic Characteristics of Mcr-1-Harboring Salmonella from Retail Meats and Eggs in Qingdao, China. *Foods* **2022**, *11*, 3854, doi:10.3390/foods11233854.
90. Perdomo, A.; Webb, H.E.; Bugarel, M.; Friedman, C.R.; Francois Watkins, L.K.; Loneragan, G.H.; Calle, A. First Known Report of Mcr-Harboring Enterobacteriaceae in the Dominican Republic. *Int. J. Environ. Res. Public. Health* **2023**, *20*, 5123, doi:10.3390/ijerph20065123.
91. Nobili, G.; La Bella, G.; Basanisi, M.G.; Damato, A.M.; Coppola, R.; Migliorelli, R.; Rondinone, V.; Leekitcharoenphon, P.; Bortolaia, V.; La Salandra, G. Occurrence and Characterisation of Colistin-Resistant Escherichia Coli in Raw Meat in Southern Italy in 2018-2020. *Microorganisms* **2022**, *10*, 1805, doi:10.3390/microorganisms10091805.
92. Kompes, G.; Duvnjak, S.; Reil, I.; Hendriksen, R.S.; Sørensen, L.H.; Zdelar-Tuk, M.; Habrun, B.; Cvetnić, L.; Bagarić, A.; Špičić, S. First Report and Characterization of the Mcr-1 Positive Multidrug-Resistant Escherichia Coli Strain Isolated from Pigs in Croatia. *Microorganisms* **2023**, *11*, 2442, doi:10.3390/microorganisms11102442.
93. Wu, S.; Cui, L.; Han, Y.; Lin, F.; Huang, J.; Song, M.; Lan, Z.; Sun, S. Characteristics, Whole-Genome Sequencing and Pathogenicity Analysis of Escherichia Coli from a White Feather Broiler Farm. *Microorganisms* **2023**, *11*, 2939, doi:10.3390/microorganisms11122939.
94. Vlad, M.-A.; Lixandru, B.-E.; Muntean, A.-A.; Trandafir, I.; Luncă, C.; Tuchiluş, C. The First Report of Mcr-1-Carrying Escherichia Coli, Isolated from a Clinical Sample in the North-East of Romania. *Microorganisms* **2024**, *12*, 2461, doi:10.3390/microorganisms12122461.
95. Kim, J.; Hwang, B.K.; Choi, H.; Wang, Y.; Choi, S.H.; Ryu, S.; Jeon, B. Characterization of Mcr-1-Harboring Plasmids from Pan Drug-Resistant Escherichia Coli Strains Isolated from Retail Raw Chicken in South Korea. *Microorganisms* **2019**, *7*, 344, doi:10.3390/microorganisms7090344.
96. Manageiro, V.; Jones-Dias, D.; Ferreira, E.; Caniça, M. Plasmid-Mediated Colistin Resistance (Mcr-1) in Escherichia Coli from Non-Imported Fresh Vegetables for Human Consumption in Portugal. *Microorganisms* **2020**, *8*, 429, doi:10.3390/microorganisms8030429.
97. Hassan, J.; Eddine, R.Z.; Mann, D.; Li, S.; Deng, X.; Saoud, I.P.; Kassem, I.I. The Mobile Colistin Resistance Gene, Mcr-1.1, Is Carried on IncX4 Plasmids in Multidrug Resistant E. Coli Isolated from Rainbow Trout Aquaculture. *Microorganisms* **2020**, *8*, 1636, doi:10.3390/microorganisms8111636.
98. Sadek, M.; Ortiz de la Rosa, J.M.; Abdelfattah Maky, M.; Korashe Dandrawy, M.; Nordmann, P.; Poirel, L. Genomic Features of MCR-1 and Extended-Spectrum β-Lactamase-Producing Enterobacterales from Retail Raw Chicken in Egypt. *Microorganisms* **2021**, *9*, 195, doi:10.3390/microorganisms9010195.

99. Moon, D.C.; Kim, S.-J.; Mechesso, A.F.; Kang, H.Y.; Song, H.-J.; Choi, J.-H.; Yoon, S.-S.; Lim, S.-K. Mobile Colistin Resistance Gene Mcr-1 Detected on an IncI2 Plasmid in Salmonella Typhimurium Sequence Type 19 from a Healthy Pig in South Korea. *Microorganisms* **2021**, *9*, 398, doi:10.3390/microorganisms9020398.
100. Teng, C.-H.; Wu, P.-C.; Tang, S.-L.; Chen, Y.-C.; Cheng, M.-F.; Huang, P.-C.; Ko, W.-C.; Wang, J.-L. A Large Spatial Survey of Colistin-Resistant Gene Mcr-1-Carrying E. Coli in Rivers across Taiwan. *Microorganisms* **2021**, *9*, 722, doi:10.3390/microorganisms9040722.
101. Zakaria, A.S.; Edward, E.A.; Mohamed, N.M. Genomic Insights into a Colistin-Resistant Uropathogenic Escherichia Coli Strain of O23:H4-ST641 Lineage Harboring Mcr-1.1 on a Conjugative IncHI2 Plasmid from Egypt. *Microorganisms* **2021**, *9*, 799, doi:10.3390/microorganisms9040799.
102. Tu, Z.; Gu, J.; Zhang, H.; Liu, J.; Shui, J.; Zhang, A. Withdrawal of Colistin Reduces Incidence of Mcr-1-Harboring IncX4-Type Plasmids but Has Limited Effects on Unrelated Antibiotic Resistance. *Pathog. Basel Switz.* **2021**, *10*, 1019, doi:10.3390/pathogens10081019.
103. Li, P.; Zhan, L.I.; Wang, H.; Gao, W.; Gao, L.; Lv, S.; Zhang, X.; Zhu, G.; Yan, Y. First Identification and Limited Dissemination of Mcr-1 Colistin Resistance in Salmonella Isolates from Jiaxing. *J. Food Prot.* **2022**, *85*, 213–219, doi:10.4315/JFP-21-069.
